# NHX Gene Family in *Camellia sinensis*: *In-silico* Genome-Wide Identification, Expression Profiles, and Regulatory Network Analysis

**DOI:** 10.3389/fpls.2021.777884

**Published:** 2021-12-20

**Authors:** Abhirup Paul, Archita Chatterjee, Shreya Subrahmanya, Guoxin Shen, Neelam Mishra

**Affiliations:** ^1^Independent Researcher, Bangalore, India; ^2^Department of Botany, St. Joseph’s College Autonomous, Bangalore, India; ^3^Sericultural Research Institute, Zhejiang Academy of Agricultural Sciences, Hangzhou, China

**Keywords:** salt tolerance, *Arabidopsis*, genome-wide search, expression profiles, *Camellia sinensis*, NHXs

## Abstract

Salt stress affects the plant growth and productivity worldwide and NHX is one of those genes that are well known to improve salt tolerance in transgenic plants. It is well characterized in several plants, such as *Arabidopsis thaliana* and cotton; however, not much is known about NHXs in tea plant. In the present study, NHX genes of tea were obtained through a genome-wide search using *A. thaliana* as reference genome. Out of the 9 NHX genes in tea, 7 genes were localized in vacuole while the remaining 2 genes were localized in the endoplasmic reticulum (ER; *CsNHX8*) and plasma membrane (PM; *CsNHX9*), respectively. Furthermore, phylogenetic relationships along with structural analysis which includes gene structure, location, and protein-conserved motifs and domains were systematically examined and further, predictions were validated by the expression analysis. The dN/dS values show that the majority of tea NHX genes is subjected to strong purifying selection under the course of evolution. Also, functional interaction was carried out in *Camellia sinensis* based on the orthologous genes in *A. thaliana*. The expression profiles linked to various stress treatments revealed wide involvement of NHX genes from tea in response to various abiotic factors. This study provides the targets for further comprehensive identification, functional study, and also contributed for a better understanding of the NHX regulatory network in *C. sinensis*.

## Background

Excessive use of inorganic fertilizers is making the land infertile and unavailable for agriculture due to over accumulation of salts in it (Kovda, 1983). Moreover, abiotic stresses, such as drought and heat stress, cause an additive effect and overall decrease the crop yield and quality (Zhu, 2001). Therefore, to keep up with growing demands of population, there is a pressing need to identify and characterize more salt tolerant genes from different plant species and use them for improvement of salt tolerance in crop plants.

Sodium chloride is one of major salts present in the soil and most of the salt tolerance mechanism focuses on the transport and compartmentalization of sodium ions. Na^+^ influx is controlled by either sodium/hydrogen antiporter (NHX) family of cation/H^+^ transporters (Apse et al., 1999) or nonselective cation channels (NSCCs), or high-affinity K^+^ transporters (HKTs; Waters et al., 2013). HKT can regulate the long-distance transport of Na^+^ (Rubio et al., 1995) while Na^+^/H^+^ antiporter (NHX) is involved in the transport of Na^+^ ions from cytoplasm to vacuole or outside of the cell. To achieve this, it utilizes the H^+^ electrochemical gradient formed by two proton pumps, i.e., H^+^-ATPase and H^+^-PPase thereby avoiding the cell from the toxic effects of sodium ions (Apse et al., 1999).

NHX proteins belong to the cation/proton antiporter 1 (CPA1) superfamily and most of NHX proteins possess 10 transmembrane helices (Yamaguchi et al., 2003; Brett et al., 2005; Chanroj et al., 2012; Wu et al., 2019b). Localization of NHX proteins is mainly restricted to plasma membranes, vacuoles, and endosomes (Aharon et al., 2003; Pehlivan et al., 2016). The first plant NHX gene was recognized in barley root tips (Ratner and Jacoby, 1976) followed by its identification and characterization in *Arabidopsis thaliana* (At; Roberto et al., 1999), and a total of 8 NHX genes have been reported in *A. thaliana* till date. Out of 8 NHXs in *A. thaliana*, 2 genes (AtNHX7 and AtNHX8) belong to PM-class (plasma membranes), 2 genes (AtNHX5 and AtNHX6) belong to Endo-class (endosomes), and 4 genes (AtNHX1-4) belong to Vac-class (vacuoles). This classification is done on the basis of their subcellular localization (Shi et al., 2000; Aharon et al., 2003; Brett et al., 2005; Bassil et al., 2011b). Apart from the involvement of these genes in salt tolerance, NHX antiporters are involved in the regulation of wide variety of physiological processes, such as vesicle trafficking, pH regulation, K^+^ homeostasis, protein transport, and growth/development (Pardo et al., 2006; Rodriguez Rosales et al., 2009; Bassil et al., 2012; Reguera et al., 2014).

*Camellia sinensis* is native to East Asia, the Indian Subcontinent, and Southeast Asia, but it is today cultivated across the world in tropical and subtropical regions. Tea plant (*C. sinensis* L.) is an important economic crop, leaves of which are an important source of non-alcoholic beverage. As a leaf-harvested crop, tea plant is unavoidably threatened with various adverse environment stresses throughout the whole life cycle, such as drought (Xie et al., 2019), salt (Wan et al., 2018), and cold (Li et al., 2018) stresses, which critically hinders the development of the tea industry. With drastic environmental changes leading to a decline in the cultivated land area, like many other economic crops, tea planting fields are moving to salinity and drought-affected areas. In this study, we performed a genome-wide analysis of NHX genes in *C. sinensis* including the phylogenetic relationships, a motif analysis, promoter analysis, gene expression pattern, and the gene structures. Through a systematic analysis of all the members of the NHX gene, we can understand the gene regulation, expression pattern, and eventually their biological functions in tea.

## Materials and Methods

### Identification of NHX Genes of Tea Plant

The tea plant genome sequence was recovered from the Tea Plant Information Archive, TPIA (Xia et al., 2019).^1^ The NHX genes from *A. thaliana* and rice were retrieved from TAIR database (Berardini et al., 2015)^2^ and Rice Genome Annotation Project database (Kawahara et al., 2013)^3^, respectively. These sequences were then used as a query sequences to scan the tea genome database using the BLASTp algorithm with an e-value of 1e-5 and an identity match of 50% as the threshold. To further confirm the presence of Na+/H+_Exchanger domain, the NHX genes were submitted to SMART (Letunic et al., 2015)^4^ and Pfam web tool. ProtParam tool integrated in ExPASy database was used to predict the physicochemical properties of the NHX peptides (Gasteiger et al., 2005).^5^ BaCello (Balanced subcellular localization predictor) online server was used to predict the subcellular localization of the protein sequences (Pierleoni et al., 2006).^6^ Additionally, TMHMM server v2.0^7^ was used to predict the transmembrane helices in NHX peptide sequences (Sonnhammer et al., 1998).

### Phylogenetic Analysis of NHX Genes

The NHX peptide sequences from *C. sinensis* (Cs), *A. thaliana* (At), *Oryza sativa* (Os), *Solanum lycopersicum* (Sl), *Solanum tuberosum* (St), *Medicago truncatula* (Mt), *Populus trichocarpa* (Pt), *Gossypium hirsutum* (Gh), *Sorghum bicolor* (Sb), *Zea mays* (Zm), and *Glycine max* (Gm) were aligned by using MUSCLE (Robert, 2004), with default parameters. The aligned sequences were then used to generate the phylogenetic tree using MEGA7.0.14 software (Kumar et al., 2016). The tree was constructed using Neighbor-Joining (NJ) algorithm with default parameters. The reliability of the phylogenetic tree was analyzed by the bootstrap method and replicates were set to 1,000.

### Conserved Motif and Gene Structure Analysis

In order to identify the conserved motifs, the MEME (Bailey et al., 2009)^8^ suite was used with default parameters. The intron/exon distribution pattern of NHX genes was obtained and then analyzed using the gene structure display server V2.0 (Hu et al., 2015).^9^

### Analysis of Cis-Regulatory Elements

The promoter sequences of 2,000 bp of the tea NHX genes were retrieved from the TPIA database to analyze the cis-acting regulatory elements (CAREs). The PlantCARE program^10^ (Rombauts et al., 1999; Lescot et al., 2002) was used for identifying and analyzing the CAREs.

### Genomic Distribution of NHX Genes and Ka/Ks Ratios

Due to the incomplete genome assembly information available in the TPIA database, the NHX genes were mapped into their corresponding scaffolds. MapGene2chromosome web v2 (MG2C) server (Jiangtao et al., 2015)^11^ was used to map the genes into their scaffolds based on their positional information in the TPIA database, which includes scaffold length, number, gene ID, starting and ending position of the genes, and scaffold ID. Further, the dN (Ka) and dS (Ks) ratios were evaluated using the SNAP v.2.1.1 online tool (Korber, 2000)^12^ to assess the synonymous and non-synonymous groups. The dS values represent the time of divergence of duplication events and the dN/dS values represent the selective pressure of duplicate genes.

### GO Ontology Annotation and Functional Interaction Network

QuickGO^13^ was used to perform GO Ontology (GO) analysis for all the 9 tea NHX genes. Furthermore, the network of functionally interacting homologous genes between tea and *A. thaliana* was identified and constructed using STRING online tool (Szklarczyk et al., 2019)^14^ with default parameters.

### Expression Profile of Tea NHXs

The tissue-specific expression profiles in 8 plant tissues, which include expression levels in apical bud, flower, fruit, young leaf, mature leaf, old leaf, root, and stem, were retrieved from TPIA database and analyzed (Xia et al., 2019). Furthermore, gene expression data under cold, drought, and salt stresses were analyzed to understand the potential role of tea NHXs in response to the abiotic stress factors. Additionally, to check the effects of methyl-jasmonate (MeJA) treatment, its expression data were retrieved from TPIA database and analyzed for the 9 tea NHXs. Respective graphs for the gene expression for all the tea NHX genes were generated. Heat maps for the same were generated using heatmapper online server (Babicki et al., 2016; Heatmapper).

## Results

### Genome-Wide Identification of NHX Genes in *C. sinensis*

In order to retrieve the members of the NHX gene family in tea, the published NHX protein sequences of *A. thaliana* (8) and rice (7) were retrieved from TAIR database (see footnote 2) and Rice Genome Annotation Project database (see footnote 3), respectively. These peptide sequences were then used as queries to search against the genome database of tea, Tea Plant Information Archive (TPIA; see footnote 1) by making use of the BLASTp algorithm with e-value and identity percentages set to 1e-5 and 50% as threshold, respectively (Supplementary Table S1). The tea NHX peptide sequences identified were further screened using the Hidden Markov Model (PF00999) to confirm the presence of the Na^+^/H^+^ _Exchanger domain. Based on the results, 9 putative tea NHX genes were incorporated into the final dataset.

The physicochemical properties of the identified tea NHX protein sequences were evaluated and analyzed by the ExPASy ProtParam tool (Table 1). The length of the NHX peptide sequences ranged from 201 (*CsNHX8*) to 1,204 (*CsNHX3*) amino acid residues while the molecular weights varied from 21764.56 (*CsNHX8*) to 134630.87 (*CsNHX3*) kDa. The predicted isoelectric points (pI) values ranged from 5.82 (*CsNHX4*) to 8.79 (*CsNHX2*). 5 out of the 9 NHX peptide sequences had more positive residues than negative ones, 3 had more of negative residues and remaining one (*CsNHX9*) had equal number of positive and negative residues. All the 9 NHX peptide sequences had positive grand average of hydropathy (GRAVY index) values, ranging from 0.209 (*CsNHX3*) to 0.695 (*CsNHX8*). This indicated that all the 9 NHX peptides identified are hydrophobic in nature. The instability index scores revealed that 2 out of 9 NHX peptides (*CsNHX2* and *CsNHX9*) were above 40 while the rest 7 had scores below the given level, indicating that most of the screened peptides had a stable nature (Wang et al., 2018). The aliphatic index of the peptides ranged from 102.02 (*CsNHX9*) to 114.38 (*CsNHX8*). The subcellular localization revealed that most of the NHX genes in tea were localized in vacuole (7 out of 9), while the remaining 2 genes were localized in the endoplasmic reticulum (ER; *CsNHX8*) and plasma membrane (PM; *CsNHX9*), respectively. Additionally, the presence of transmembrane helices was also analyzed and it revealed that all the NHX peptides had a considerable number of transmembrane helices, ranging from a minimum of 6 in *CsNHX8* to a maximum of 12 in *CsNHX9* (Supplementary Figure S1).

**Table 1:** Sequence characteristics and physicochemical properties of NHX genes in *Camellia sinensis*.

Gene ID	Gene name	Locus position	Gene length (bp)	Protein length (aa)	Mol. Wt. (KD)	pI value	No. of negative residues	No. of positive residues	GRAVY index	Instability index	Aliphatic index	Subcellular localization
TEA012938.1	CsNHX1	Scaffold1720: 567706− 577760+	10,054	541	59727.27	8.39	32	35	0.569	39.05	110.07	Vacuole
TEA012286.1	CsNHX2	Scaffold338: 1074858− 1067174−	7,684	541	59731.31	8.79	31	36	0.574	40.63	110.81	Vacuole
TEA021179.1	CsNHX3	Scaffold2776: 688282− 666811+	21,471	1,204	134630.87	6.19	126	118	0.209	35.28	105.18	Vacuole
TEA012245.1	CsNHX4	Scaffold7171: 367289− 358099−	9,190	535	58454.29	5.82	40	33	0.561	37.68	108.75	Vacuole
TEA000661.1	CsNHX5	Scaffold4401: 2616944− 2627254+	10,310	493	54483.76	8.44	34	38	0.473	35.92	105.54	Vacuole
TEA025916.1	CsNHX6	Scaffold3845: 424226− 432801+	8,575	541	59633.63	7.58	39	40	0.588	34.89	112.62	Vacuole
TEA023041.1	CsNHX7	Scaffold1950: 942195− 925240−	16,955	498	56333.95	8.76	38	43	0.441	35.13	103.07	Vacuole
TEA011468.1	CsNHX8	Scaffold5365: 135491− 139599+	4,108	201	21764.56	6.04	13	11	0.695	38.90	114.38	ER
TEA006997.1	CsNHX9	Scaffold2078: 1418352− 1496831+	78,479	1,156	128350.42	7.38	113	113	0.064	42.54	102.02	Plasma membrane

*Locus position, gene length, protein length, molecular weight and pI value, no. of negative and positive residues, GRAVY index, instability index, aliphatic index, and subcellular localizations were analyzed*.

### Phylogenetic Analysis of Tea NHXs

To explore the evolutionary relationships of the NHX genes among the different plant species, a phylogenetic analysis was conducted comparing the identified tea NHX genes along with NHXs from 10 other plants. For this study, we retrieved the NHX peptide sequences from *A. thaliana* (At), *O. sativa* (Os), *S. lycopersicum* (Sl), *S. tuberosum* (St), *M. truncatula* (Mt), *P. trichocarpa* (Pt), *G. hirsutum* (Gh), *S. bicolor* (Sb), *Z. mays* (Zm), and *G. max* (Gm) from their respective genome databases. The NHX peptide sequences from *A. thaliana* were used as query sequences to search for the NHX genes in all these plants. The sizes of all the NHX gene family from the 11 members ranged from a minimum of 5 in *S. tuberosum* to a maximum of 23 in *G. hirsutum* (Table 2).

**Table 2:** NHX gene family members from *A. thaliana* (At), *C. sinensis* (Cs), *O. sativa* (Os), *S. lycopersicum* (Sl), *S. tuberosum* (St), *M. truncatula* (Mt), *P. trichocarpa* (Pt), *G. hirsutum* (Gh), *S. bicolor* (Sb), *Z. mays* (Zm), and *G. max* (Gm).

Class\Plants	At	Cs	Os	Sl	St	Mt	Pt	Gh	Sb	Zm	Gm
Vac	4	7	4	7	5	6	5	17	4	4	8
Endo	2	1	2	0	0	1	1	4	2	2	3
PM	2	1	1	0	0	0	2	2	1	1	1
Total	8	9	7	7	5	7	8	23	7	7	12

*The gene family has been grouped into 3 different subfamilies based on their subcellular localizations*.

**Figure 1:**
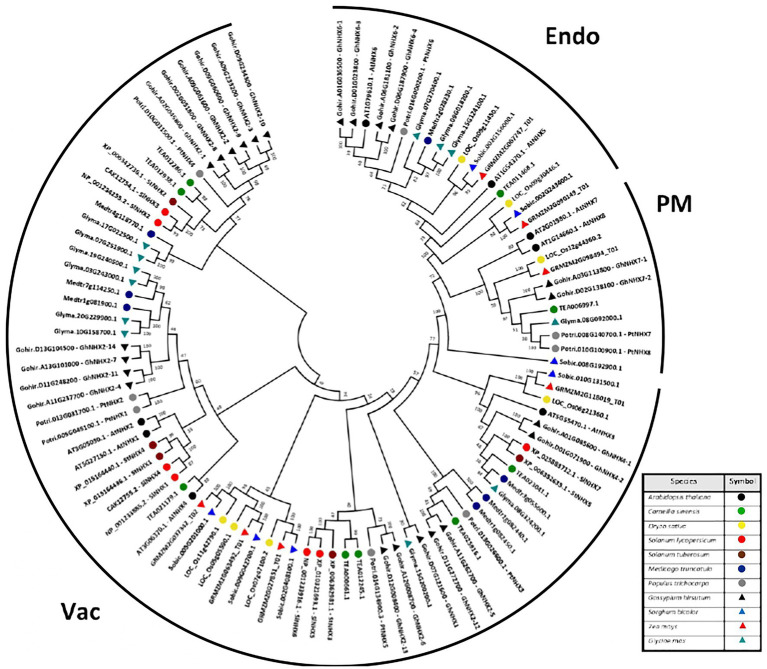
Phylogenetic tree of NHX genes from *Arabidopsis thaliana*, *Camellia sinensis*, *Oryza sativa*, *Solanum lycopersicum*, *Solanum tuberosum*, *Medicago truncatula*, *Populus trichocarpa*, *Gossypium hirsutum*, *Sorghum bicolor*, *Zea mays*, and *Glycine max*. The full-length NHX protein sequences were aligned using MUSCLE, and the phylogenetic tree was constructed using MEGA 7.0.14 by the Neighbor-Joining (NJ) method with default parameters and 1,000 bootstrap replicates. The tree is divided into three major classes of NHX genes, consisting of the Vac-, Endo-, and PM-classes.

The phylogenetic tree was then constructed using all the 100 NHX peptide sequences from the 11 species. MEGA 7.0.14 was used to generate the phylogenetic trees, using the Neighbor-Joining (NJ) algorithm, at default parameters and 1,000 bootstrap replicates. The phylogenetic tree shows a direct relation with the subcellular localization as all the NHX peptides clustered into 3 different clades based on their localizations (Figure 1). The 3 different clades were the Vac-class (Vacuole), Endo-class (Endosomal), and PM-class (Plasma membrane). Among these 3 classes, the Vac-class was the most abundantly present class of NHXs in all the 11 species with 71 genes, followed by the Endo-class and PM-class with 18 and 11 genes, respectively.

### Motif Composition of Tea NHXs

**Figure 2:**
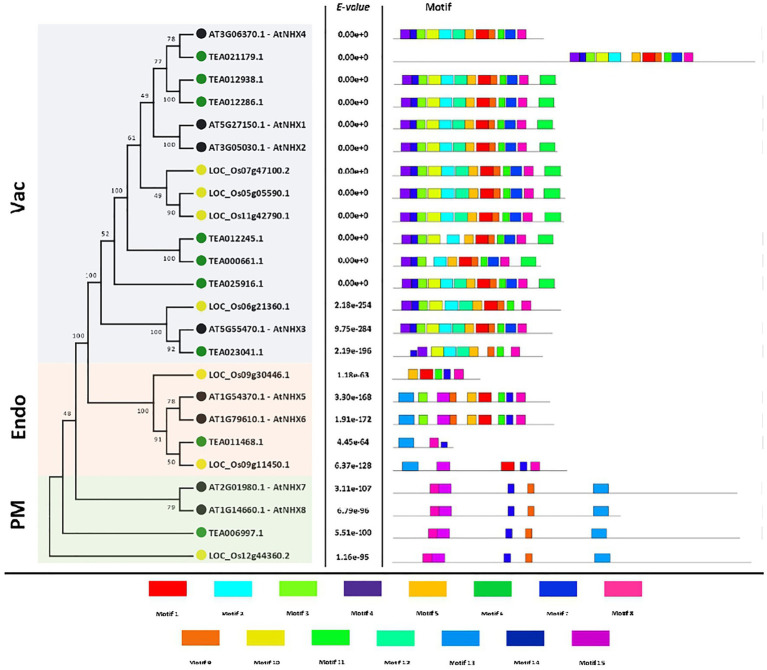
The motif analysis of NHX genes in *A. thaliana*, *C. sinensis*, and *O. sativa*. The motif figures were generated by MEME suite. A total of 15 motifs were identified and are marked individually.

To evaluate the structural characteristics and diversity of the tea NHXs, a correlative study of the conserved motifs from the NHX peptides of *A. thaliana*, *C. sinensis*, and *O. sativa* was conducted using the MEME suite (Figure 2). 15 motifs were identified from 24 NHXs used out of which 2 (Motif 8 and Motif 14) were conserved across all the genes. Motif 1, 5, and 11 were each present in 18 NHXs. These 3 motifs existed only in the Vac- and Endo-classes. The amiloride-binding site (FFIYLLPPI) is a characteristic feature of NHX proteins. It was detected in Motif 3 and was found in 16 NHXs, existing only in the Vac- and Endo-classes. Motif 2, 4, 6, 10, and 12 existed only in the Vac-class and was present in 15, 15, 10, 14, and 12 NHXs, respectively. Motif 13 and 15 were present in 8 and 7 NHXs correspondingly. These 2 motifs existed only in the PM- and Endo-classes. The remaining motif 7 and 9 were present in all the classes and were harbored by 22 and 21 NHXs correspondingly. Additionally, the motif logos of all the 15 motifs were also obtained and are presented in the supplemental information (Supplementary Figure S2). The NHXs present in the same class had similar conserved motifs except Endo-class, which showed partial conservancy. These results provided noteworthy evidence that the NHX genes were highly conserved.

### Gene Structure Analysis of Tea NHXs

**Figure 3:**
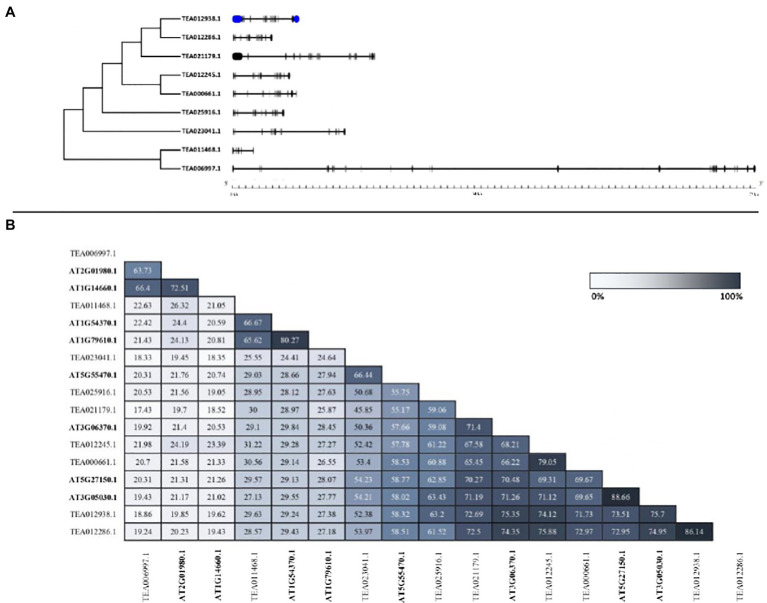
The intron/exon architecture and pairwise sequence identity among NHX proteins of *A. thaliana* and *C. sinensis*. **(A)** Gene structure maps were drawn using the Gene Structure Display Server 2.0. Black boxes represent exons, blue boxes represent the UTRs, and black lines represent introns. The gene length can be estimated by using the scale (in kb) given at the bottom. **(B)** The full-length protein sequences were aligned using MUSCLE tool (https://www.ebi.ac.uk/Tools/msa/muscle/) with default settings. The table has been marked based on a gradient with lighter shades representing minimum percentage identity and darker shades representing maximum identity between the sequences.

To identify the structural characteristics of the tea NHXs, the intron/exon architecture of the genes were analyzed using Gene Structure Display Server v2.0. Study of the intron/exon patterns revealed some significant differences concerning the number of introns and exons, which further contributes to the variation in gene lengths. Abundant presence of non-coding sequences within a genome is regarded to be an indicator of genome complexity (Taft et al., 2007; Goyal et al., 2018; Chatterjee et al., 2020). Analyzing these intron arrangements thereby provides significant information regarding the evolution, regulation, and function of the NHXs (Deutsch and Long, 1999; Fedorova and Fedorov, 2003; Zhang et al., 2014; Liu et al., 2015). The analysis of the tea NHX gene structures indicated considerable differences with respect to the number of introns and exons across the 3 classes (Figure 3A). Among the 9 tea NHXs, only *CsNHX1* (TEA012938.1) possessed UTR (Untranslated Regions) segments at both 5′ and 3′ ends. 5 out the 7 Vac-class NHXs had 14 exons and 13 introns. *CsNHX3* (TEA021179.1) possessed 19 exons and 18 introns while *CsNHX7* (TEA023041.1) had 13 exons and 12 introns, respectively. The Endo-class NHX *CsNHX8* (TEA011468.1) had the least share of IEs (Introns-exons) among the 3 classes with only 6 exons and 5 intron segments. However, the PM-class NHX *CsNHX9* (TEA006997.1) had the most share of IEs with 25 exons and 24 introns. It was observed that the genes belonging to the same clade had a similar distribution of introns and exons. The intron segments and exon lengths were relatively conserved among the genes of the same class. Additionally, analyzing the amino acid sequence identity also supported the sequence conservation among the tea NHXs (Figure 3B). Two paralogous pairs of NHX in Vac-class displayed high amino acid sequence identities (TEA012286.1/TEA012938.1 = 86.14% and TEA012245.1/TEA000661.1 = 79.05%). On the flip side, tea NHXs in different classes displayed lower levels of sequence identities (TEA012245.1/TEA006997.1 = 21.98%; TEA012245.1/TEA011468.1 = 31.22%; and TEA011468.1/TEA006997.1 = 22.63%).

### Retrieval of Tea NHX Promoter Regions and Analysis of CAREs

**Figure 4:**
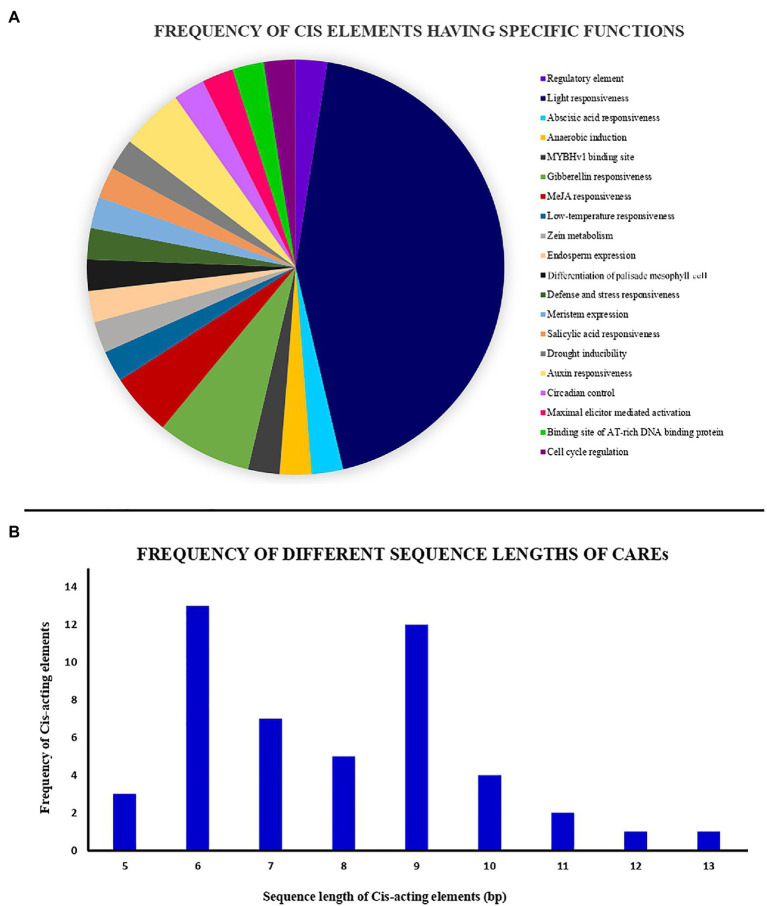
Analysis of cis-acting elements identified from the NHX genes of *C. sinensis*. All cis-acting elements have been identified using PlantCARE database. **(A)** Pie chart showing the frequency of different cis-acting elements based on their specific biological activities. **(B)** Histogram showing the frequency of different sequence lengths of the cis-acting elements.

Cis-acting regulatory elements (CAREs) play a key role in determining gene regulation, function, transcription, and gene expression (Wu et al., 2019a; Liu et al., 2021). Analysis of these regulatory elements helps in defining the plant responses to various environmental stimuli, stress factors, thereby affecting the growth regulation (Akram et al., 2020). To explore the transcriptional potential of the tea NHX genes, the promoter sequences of 2,000 bp upstream of the transcriptional start codon “ATG” were retrieved from the TPIA database. These promoter sequences were then used to predict and analyze the CAREs using the PlantCARE database. 41 total CAREs were identified randomly distributed across the promoter regions of the 9 tea NHXs (Supplementary Table S2). Based on the specific biological functions of the identified CAREs, they were grouped together into a pie chart under 20 different sections (Figure 4A). Most of the CAREs had sequence lengths of 6 and 9 bp, while the others ranged between 5 to 13 bp (Figure 4B). Analyzing the 41 CAREs, it was observed that 18 elements were involved in light responsiveness, 9 elements in phyto-hormonal as well as plant growth and regulation each, and 5 elements in stress response. The light responsive elements had the largest share of CAREs and were present in all the 9 tea NHXs. Among these 18 light responsive elements, the Box-4 and G-box elements were abundantly present in 8 and 6 NHXs, respectively. Few of the other light responsive elements were TCCC-motif, AE-box, AT1-motif, Box-II, TCT-motif, chs-CMA1a, and chs-CMA2a in 4, 2, 2, 1, 1, 3, and 3 tea NHXs, respectively. NHXs are mostly involved in response to various environmental stresses and regulation (Akram et al., 2020). The stress responsiveness elements comprised of elements responding to drought stress (MBS), low temperature (LTR), defense and stress (TC-rich repeats), and anaerobic induction (ARE) in 1, 2, 3, and 7 tea NHXs correspondingly. Another element was involved in maximal elicitor mediated activation (AT-rich sequences) was harbored by 1 tea NHX (TEA023041.1). The CAREs involved in phytohormone responses mainly comprised of abscisic acid responsive element (ABRE), gibberellin responsive elements (GARE-motif, TATC-box, and P-box), and salicylic acid responsive element (TCA-element) in 6, 5, and 5 genes, respectively. Other phytohormone response elements included elements responsive to MeJA (CGTCA-motif and TGACG-motif) in 4 genes and auxin (TGA-element and AuxRR-core) in 2 genes. The elements associated with plant growth and development mainly comprised of MYBvH1-binding site (CCAAT-box), zein metabolism regulatory element (O2-site), endosperm expression element (GCN4_motif), and palisade mesophyll differentiation element (HD-Zip 1) in 5, 4, 3, and 3 tea NHXs, respectively. A regulatory element (A-box) was present in *CsNHX1* (TEA012938.1) and *CsNHX9* (TEA006997.1), while AT-rich DNA-binding site (ATCT-motif) was present in *CsNHX8* (TEA011468.1) and *CsNHX9* (TEA006997.1). Some of the other growth-related CAREs included elements involved in meristem expression (CAT-box) and circadian control, both present in *CsNHX5* (TEA000661.1) and cell cycle regulation (MSA-like) in *CsNHX8*. The results obtained from the analysis of these CAREs suggests the involvement of the tea NHXs in various phytohormone, light, and stress responses.

### Genomic Distribution Map and Evolutionary Pressures on Tea NHXs

**Figure 5:**
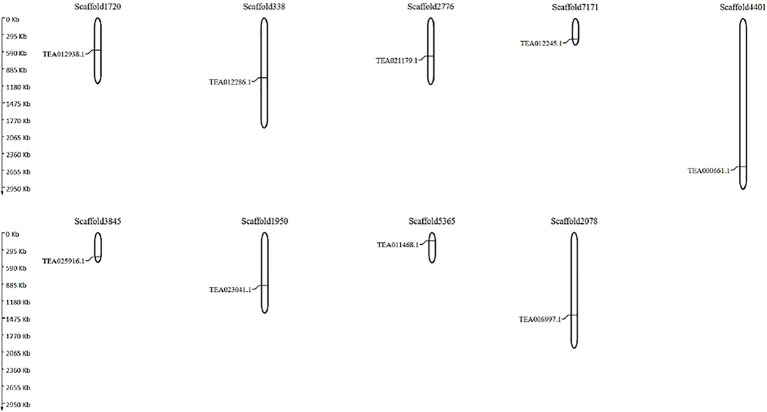
The scaffold distribution 9 NHX genes in *C. sinensis*. MapGene2chromosome web v2 (MG2C) software tool (http://mg2c.iask.in/mg2c_v2.1/) was used to map genes into their respective scaffolds. The scaffolds are drawn to scale and the scaffold numbers are indicated on the top.

In an attempt to understand the genome distribution pattern of the tea NHXs, the genes were mapped into their genomic scaffolds. Due to the lack of chromosome-level assembly data in the TPIA database, the genes had to be mapped into their scaffolds instead of the chromosomes. The 9 tea NHXs were distributed evenly across 9 different scaffolds (Figure 5). The genes were positioned such that a single scaffold housed individual genes. Additionally, the Ka/Ks or dN/dS (non-synonymous substitution rate/synonymous substitution rate) ratios were calculated in order to understand the evolutionary pressures and gene divergence mechanisms (Supplementary Table S3). The dN/dS ratio helps determine whether Darwinian selection pressures were involved in the duplication events (Tian et al., 2017; Chatterjee et al., 2020). If the value of the dN/dS ratio is >1, it implies a positive or Darwinian selection. If the ratio is equal to 1, it implies a neutral selection and if the ratio is <1, it determines a purifying selection (Bowers et al., 2003; Liu et al., 2014). Pairwise comparisons of the 9 tea NHXs revealed 13 gene pairs having their dN/dS ratios >1, indicating a positive selection. The rest 23 gene pairs had their ratios <1, indicating a negative or purifying selection. Additionally, a cumulative graph of the tea NHXs was also generated (Supplementary Figure S3). The results from the gene distribution pattern and dN/dS ratios showed that the NHXs were extensively distributed across the *C. sinensis* genome. Tandem duplication events were however absent across the tea NHXs. The dN/dS ratios are conclusive proof that strong purifying selection pressures had occurred during the evolution thereby enabling a number of different factors to regulate the NHXs in tea genome.

### GO Ontology Analysis of Tea NHXs

**Figure 6:**
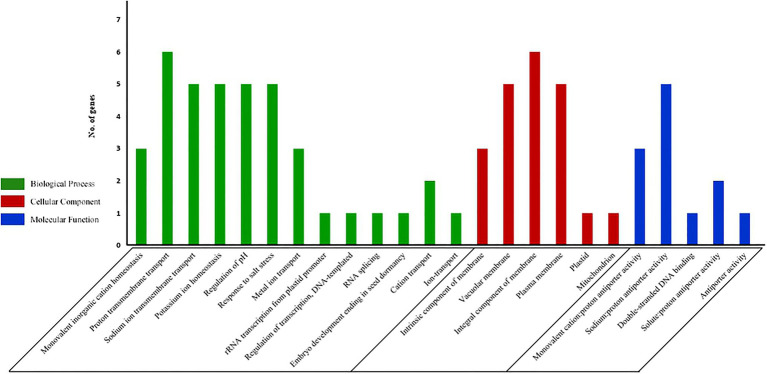
GO analysis of all the 9 NHX genes in *C. sinensis*. The results have been grouped into three main categories: Biological Process, Cellular Component, and Molecular function. The y-axis represents the frequency of genes while the x-axis represents the potential functions.

In order to predict the functions of the 9 tea NHXs, GO ontology analysis was done. It was observed that tea NHXs were enriched in 24 GO terms (Supplementary Table S4). The 9 NHX genes were divided into 3 major groups, which included biological process, cellular component, and molecular function. The first group featured 13 different GO terms with “proton transmembrane transport” (GO:1902600; 6 sequences; 66.67%) having the highest representation (Figure 6). It was followed by “Na^+^ transmembrane transport” (GO:0035725; 5 sequences; 55.56%), “K^+^ homeostasis” (GO:0055075; 5 sequences; 55.56%), “regulation of pH” (GO:0006885; 5 sequences; 55.56%), and “response to salt” (GO:0009651; 5 sequences; 55.56%). Few of the other GO terms included “monovalent inorganic cation homeostasis” (GO:0055067), “metal ion transport” (GO:0030001), and “RNA splicing” (GO:0008380). The first group was followed by the cellular component group that featured 6 different GO terms. Among these 6, “integral component of membrane” (GO:0016021; 6 sequences; 66.67%) was featured the most and was closely followed by “vacuolar membrane” (GO:0005774; 5 sequences; 55.56%) and “plasma membrane” (GO:0005886; 5 sequences; 55.56%). The rest was “intrinsic component of membrane” (GO:0031224; 3 sequences; 33.33%), “plastid” (GO:0009536; 1 sequence; 11.11%), and “mitochondria” (GO:0005739; 1 sequence; 11.11%). The remaining 5 of the 24 identified GO terms were featured in the molecular function group. Among these 5, “Na:proton antiporter activity” (GO:0015386; 5 sequences; 55.56%) was represented the most. It was followed by “monovalent cation:proton antiporter activity” (GO:0005451; 3 sequences; 33.33%), “solute:proton antiporter activity” (GO:0015299; 2 sequences; 22.22%), “double stranded DNA binding” (GO:0003690; 1 sequence; 11.11%), and “antiporter activity” (GO:0015297; 1 sequence; 11.11%).

### Functional Interaction Network of Tea NHX Proteins

**Figure 7:**
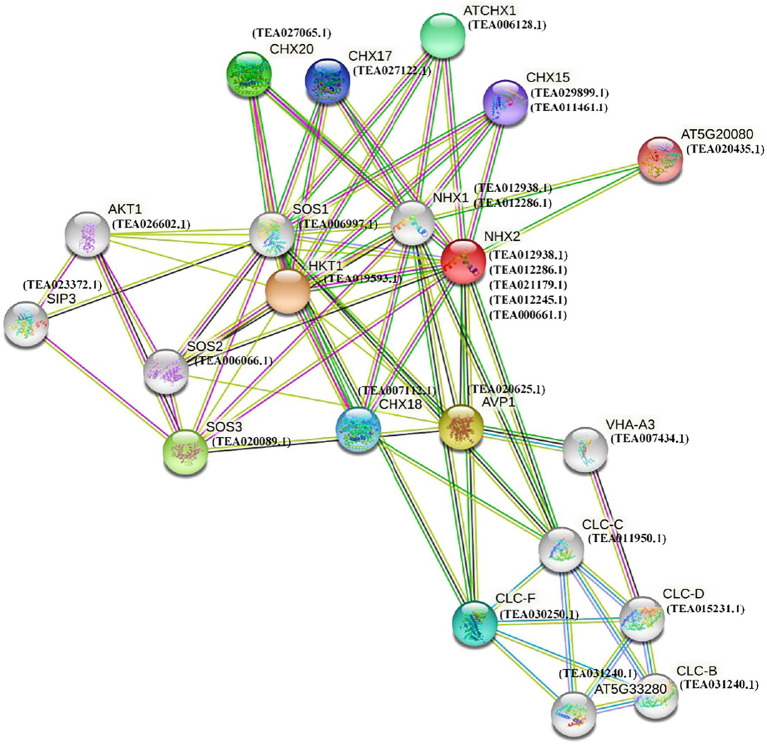
Functional interaction networks of NHX proteins in *C. sinensis*. The interaction network was formed according to homologs in *A. thaliana*.

To understand and explore the interaction pattern of NHX genes in tea, a protein interaction network was constructed using the STRING server based on an *Arabidopsis* association model (Figure 7). The *Arabidopsis* model had to be employed due to the absence of tea database in the STRING server. NHXs are largely involved in a variety of biological processes of which response to salt stress is one of the prominent ones (Tian et al., 2017). The interaction network was therefore constructed based on 5 tea NHXs (*CsNHX1*, *CsNHX2*, *CsNHX3*, *CsNHX4*, and *CsNHX5*), involved in response to salt stress according to GO ontology. The *A. thaliana* homolog for these 5 tea NHXs, AtNHX2 (AT3G05030), was used as the central node to build the full network. The tea proteins, homologs to the *Arabidopsis* proteins participating in the network, were also added. These homologous proteins were designated as STRING proteins and were selected based on high bit scores in BLAST results (Chatterjee et al., 2020). Similarity search program, such as BLAST, is frequently used to produce accurate statistical estimates that help ensuring protein sequences with significant similarity to have similar structures (Pearson, 2013). In addition, proteins sharing higher degree of sequence and structural similarities often tend to have similar functions as well (Gan et al., 2002). AtNHX2 (AT3G05030) is involved in active K^+^ uptake at the tonoplast and involved in regulating stomatal closure (Berardini et al., 2015). AtNHX1 (AT5G27150) encodes a vacuolar sodium-proton antiporter involved in salt tolerance, ion homeostasis, and leaf development. Two of the tea NHXs (*CsNHX1* and *CsNHX2*) which are homologous to AtNHX1 are massively involved in response to salt stress. AVP1 (AT1G15690) is involved in regulation of apoplastic pH and auxin transport (Berardini et al., 2015). CLC-C (AT5G49890) is a chloride channel protein and is involved in the Cl^−^ transmembrane transporter activity. CLC-F (AT1G55620) is another chloride channel protein, localized in chloroplast and Golgi apparatus, and is involved in voltage-gated Cl^−^ channel activity (Berardini et al., 2015). HKT1 (AT4G10310) encodes for a sodium transporter expressed in xylem parenchyma cells and is involved in response to osmotic stress and salt stress. SOS2 (AT5G35410) encodes a member of the CBL-interacting protein kinase family and is a regulatory component controlling plant K^+^ nutrition (Berardini et al., 2015). TEA006066.1, which is homologous to SOS2, also shows response to nutrient and water deprivation according to GO ontology. SOS3 (AT5G24270) encodes for a calcium sensor that is essential for K^+^ nutrition, K^+^/Na^+^ selectivity, and salt tolerance. CHX18 (AT5G41610), CHX17 (AT4G23700), and CHX15 (AT2G13620) are all involved in regulation of pH and are members of the putative Na^+^/H^+^ antiporter family (Berardini et al., 2015).

### Tissue-Specific Gene Expression of Tea NHXs

**Figure 8:**
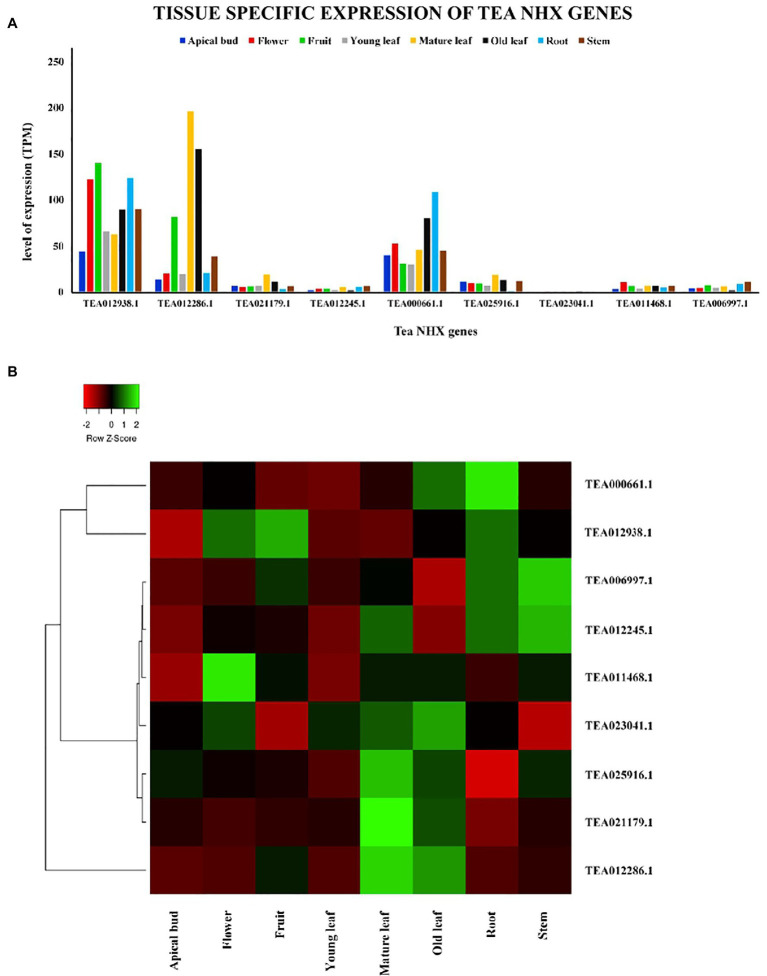
Tissue-specific expression patterns of NHX genes in *C. sinensis* in 8 different plant tissues. **(A)** The relative expression of Tea NHX genes represented graphically by analyzing the transcriptome data. **(B)** Relative expression represented as a heatmap, generated using heatmapper online server. The color bar on the top represents the normalized transcript per million (TPM) values. Green and red colors represent the up- and downregulation values while black represents no expression.

The tissue-specific expression levels of the 9 tea NHXs in 8 different tissues were retrieved from the TPIA database wherein the levels of expressions were evaluated in transcripts per million (TPM). The database has the expression profile data of all the *C. sinensis* genes, which have been experimentally validated (Wei et al., 2018). The plant tissues that have been assessed in the study involved apical bud, flower, fruit, young leaf, mature leaf, old leaf, root, and stem (Supplementary Table S5). All the 9 tea NHXs exhibited varying levels of expression in these 8 different tissues. Few of the genes had high levels of expression while the rest had negligible transcript levels (Figure 8). *CsNHX1* (TEA012938.1) was expressed the most in apical bud, closely followed by *CsNHX5* (TEA000661.1). This similar pattern was observed when the expression levels were checked in flower, young leaf, root, and stem. The highest expression level was recorded by *CsNHX1* in fruit, followed by *CsNHX2* (TEA012286.1) and *CsNHX5* (TEA000661.1). In mature leaf and old leaf, *CsNHX2* was expressed the most, followed by *CsNHX1* and *CsNHX5*. These results suggested that 3 out of the 9 tea NHXs (*CsNHX1*, *CsNHX2*, and *CsNHX5*) were significantly expressed in all the 8 tissues. The rest of the 6 NHXs were minimally expressed in these 8 tissues with *CsNHX7* (TEA023041.1) and *CsNHX4* (TEA012245.1) being the least.

### Expression Profiles of Tea NHXs Under Cold and Drought Stress

**Figure 9:**
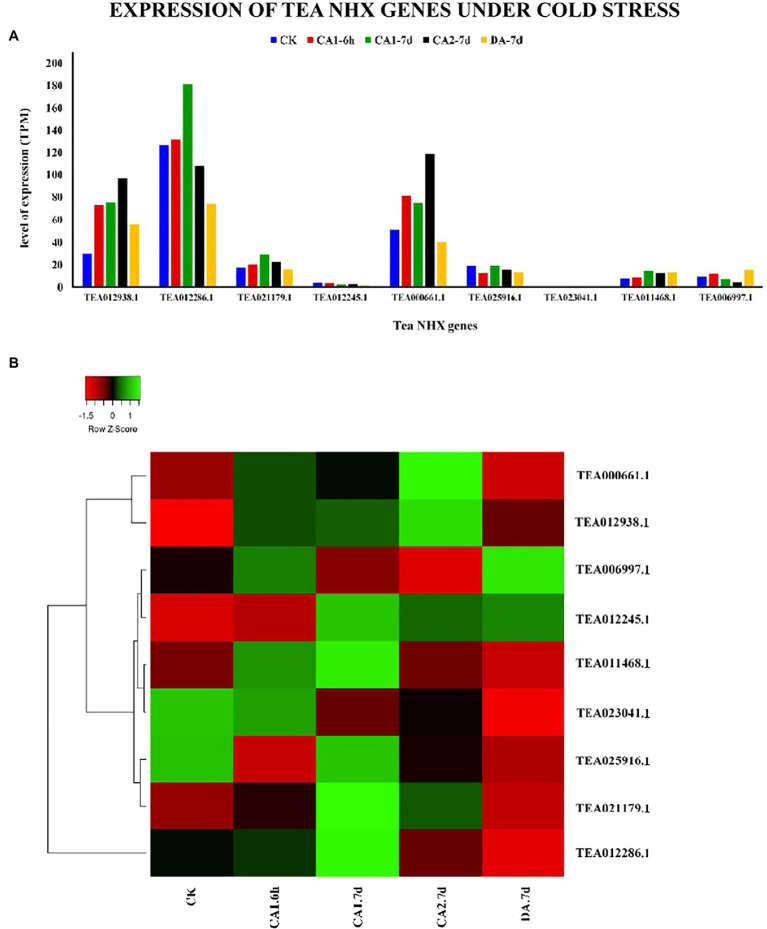
Expression of NHX genes in *C. sinensis* under cold stress. **(A)** The relative expression of Tea NHX genes represented graphically by analyzing the transcriptome data. **(B)** Relative expression represented as a heatmap, generated using heatmapper online server. The color bar on the top represents the normalized TPM values. Green and red colors represent the up- and downregulation values while black represents no expression.

**Figure 10:**
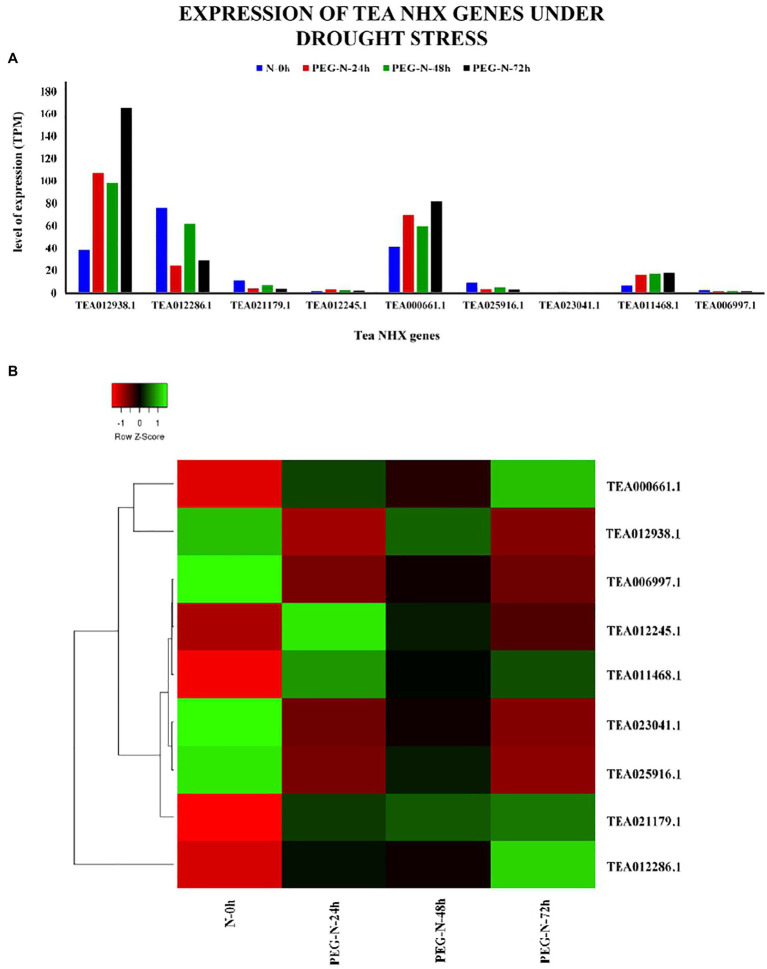
Expression of NHX genes in *C. sinensis* under drought stress. **(A)** The relative expression of Tea NHX genes, represented graphically by analyzing the transcriptome data. **(B)** Relative expression represented as a heatmap, generated using heatmapper online server. The color bar on the top represents the normalized TPM values. Green and red colors represent the up- and downregulation values, respectively, while black represents no expression.

In order to check how the 9 tea NHXs respond to varying levels of cold and drought stress, their expression data were retrieved from the TPIA database. The TPIA database has experimentally verified expression data for all the *C. sinensis* genes under cold (Wang et al., 2013) and drought stress (Zhang et al., 2017). The cold acclimated data comprised of 5 stages of expression: (1) 25~20°C (CK), (2) Fully acclimated at 10°C for 6 h (CA1-6 h), (3) 10~4°C for 7 days (CA1-7 d), (4) Cold response at 4~0°C for 7 days (CA2-7 d), and (5) Recovering under 25~20°C for 7 days (DA-7 d; Xia et al., 2019), where CK is the control (Supplementary Table S6). Expression levels for CA1-6 h showed that 7 out of 9 tea NHXs were upregulated while the rest 2 were downregulated. Out of these 7 upregulated genes, *CsNHX1* (TEA012938.1), *CsNHX5* (TEA000661.1), and *CsNHX2* (TEA012286.1) were upregulated the most. When the cold stress was increased to the next stage (CA1-7 d), again 7 genes showed upregulation with *CsNHX2*, *CsNHX1*, and *CsNHX5* being the highest. *CsNHX9* (TEA006997.1), which was initially upregulated in the first condition, was downregulated in this present condition. Further increasing the cold stress levels at CA2-7 d, expression data revealed 5 NHXs being upregulated. *CsNHX6* (TEA025916.1) was slightly upregulated at CA1-7 d but was downregulated at CA2-7 d. *CsNHX2* was also upregulated for the previous two levels of cold stress, but at CA2-7 d, it was downregulated. *CsNHX5* was expressed the most followed by TEA12938.1. Expression levels under the recovery phase (DA-7 d) showed that only 3 NHXs were upregulated (Figure 9). Throughout the cold stress conditions, 2 genes namely, *CsNHX1* and *CsNHX5* consistently maintained high levels of expression, followed by *CsNHX2* and *CsNHX3*. These results indicated the active participation of these 4 tea NHX genes in response to cold stress. The expression levels were further checked under drought stress. The expression data in the TPIA database with respect to 25% polyethylene glycol (PEG) treatment include four stages: (1) 0 h, (2) 24 h, (3) 48 h, and (4) 72 h (Zhang et al., 2017), where 0 h was taken as the control (Supplementary Table S7). Under the first drought stress period of 24 h, 5 of the 9 tea NHXs were upregulated with *CsNHX1* being expressed the most. The same set of genes was upregulated when the drought stress condition was extended to a period of 48 h and then for 72 h (Figure 10). These 5 genes showed upregulated levels of expression throughout the experimental conditions and thereby suggest their roles in response to drought stress.

### Expression Profiles of Tea NHXs Under Salt Stress

**Figure 11:**
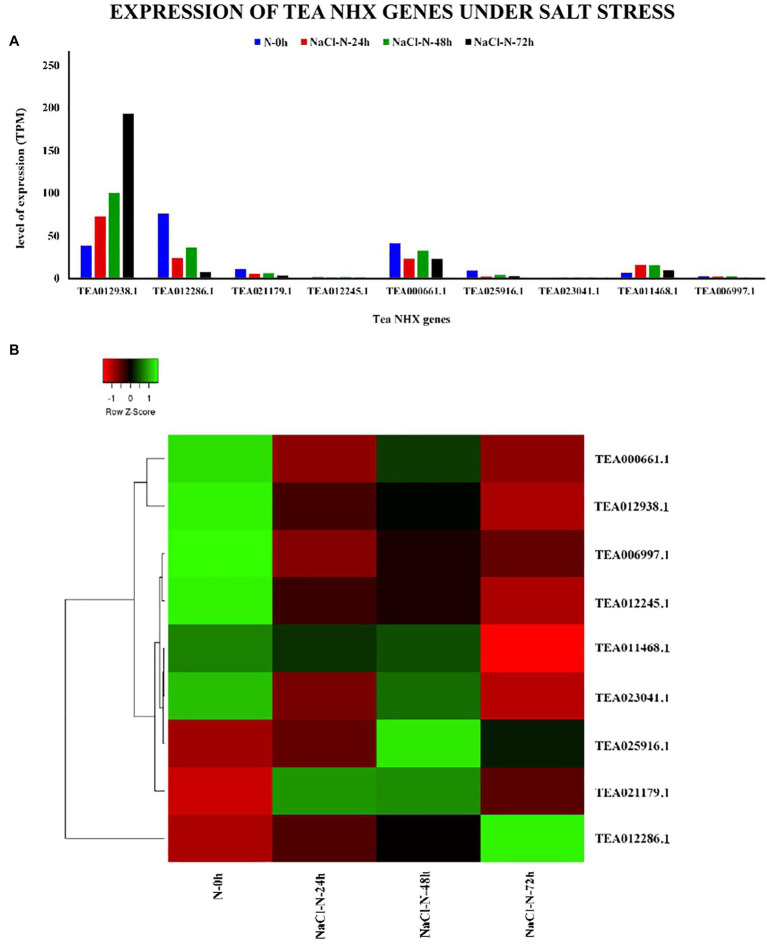
Expression of NHX genes in *C. sinensis* under salt stress. **(A)** The relative expression of Tea NHX genes, represented graphically by analyzing the transcriptome data. **(B)** Relative expression represented as a heatmap, generated using heatmapper online server. The color bar on the top represents the normalized TPM values. Green and red colors represent the up- and downregulation values while black represents no expression.

The primary role of the NHX genes is response to salt stress (Tian et al., 2017). To understand the potential role of the 9 tea NHXs in response to high levels of salinity, the expression data were analyzed. The salt stress data in TPIA database are recorded based on treatment with 200 mm NaCl under 4 stages: (1) 0 h, (2) 24 h, (3) 48 h, and (4) 72 h (Zhang et al., 2017) where 0 h was taken as the control (Supplementary Table S8). Expression data under the 24 h salt stress condition revealed 3 genes being upregulated. Among these 3 tea NHXs, *CsNHX1* (TEA012938.1) was expressed the most. A similar pattern was observed when the salt stress conditions were extended for periods of 48 h and 72 h (Figure 11). *CsNHX7* (TEA023041.1) and *CsNHX8* (TEA011468.1) were upregulated to a fair extent while *CsNHX1* maintained very high levels of expression throughout the experimental condition, with increasing transcript levels at each stage. GO ontology data too suggested the involvement of *CsNHX1* in response to salt stress. These results clearly indicate the active role of these tea NHXs in response to prolonged levels of salt stress.

### Response of Tea NHXs to MeJA Treatment

**Figure 12:**
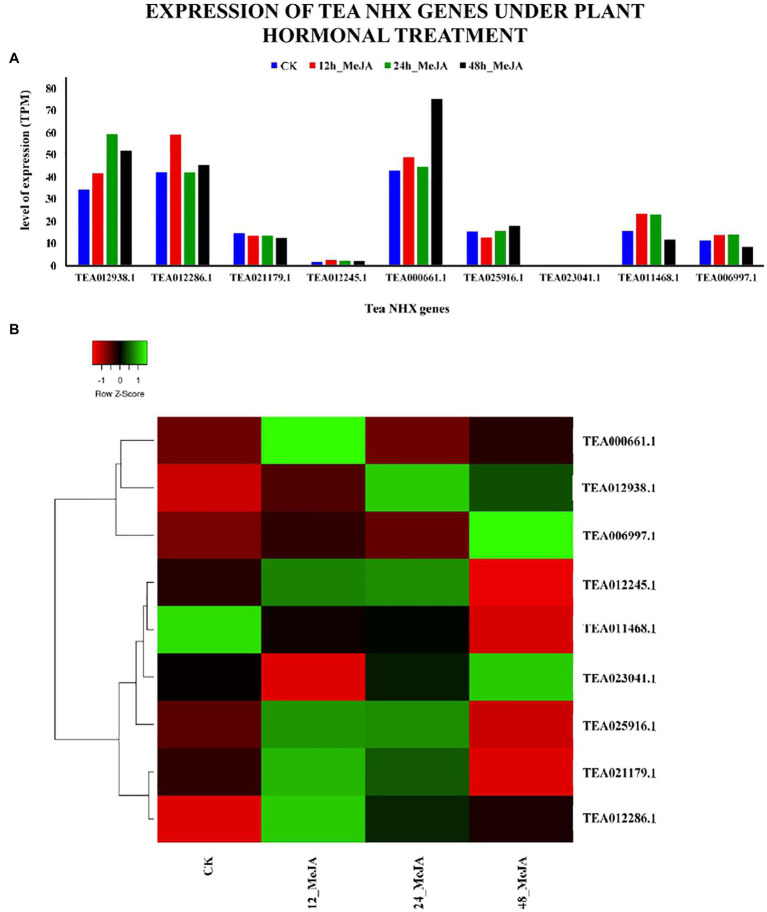
Expression levels of NHX genes in *C. sinensis* under plant hormonal treatment. The plant hormone under study was Methyl-jasmonate (MeJA). **(A)** The relative expression of Tea NHX genes, represented graphically by analyzing the transcriptome data. **(B)** Relative expression represented as a heatmap, generated using heatmapper online server. The color bar on the top represents the normalized TPM values. Green and red colors represent the up- and downregulation values while black represents no expression.

The analysis of the cis-acting elements in the promoter regions of the 9 tea NHXs had revealed the presence of 2 MeJA responsive elements (CGTCA-motif and TGACG-motif; Supplementary Table S2). To further understand the effect of MeJA on the 9 tea NHXs, their expression data were retrieved from the TPIA database and analyzed. This data is recorded based on the results of exposing the plant parts to aqueous solution of MeJA, under 4 stages: (1) 0 h, (2) 12 h, (3) 24 h, and (4) 48 h (Shi et al., 2015) where, 0 h was used as the control (Supplementary Table S9). 7 out of 9 tea NHXs showed upregulation in expression levels when exposed to the MeJA treatment for a period of 12 h. Extending the duration of the experiment to 24 h showed a few minor changes in the genes showing upregulation. *CsNHX2* (TEA012286.1), which was showing upregulation initially, now was slightly downregulated. On the other hand, *CsNHX6* (TEA025916.1) was downregulated in the initial phase but showed upregulated levels of expression in the present condition. 7 genes were upregulated in total at 24 h of MeJA treatment. Further extending the experiment to 48 h revealed that 5 genes were upregulated while the rest 4 were downregulated (Figure 12). *CsNHX1* (TEA012938.1) and *CsNHX5* (TEA000661.1) consistently maintained high levels of expression throughout the 48 h of the exposure to MeJA. These results suggested that the transcription levels of the tea NHXs might have a close relation to the regulation of MeJA.

## Discussion

NHX gene families have already been identified and functionally characterized for several plants, including *A. thaliana*, rice, wheat, sweet beet, cotton, and other (Kumari et al., 2018; Yarra, 2019; Wu et al., 2019b; Fu et al., 2020; Khare et al., 2021). However, the NHX genes in *C. sinensis* have not been studied yet. In this study, the gene structure, phylogenetic relationship, genomic distribution, and expression of NHX genes in *C. sinensis* were all analyzed at the genomic level. A diverse no. of NHX genes have been identified in various plant species. Gene duplication and loss specific to different subfamilies of NHX over the course of evolution could explain these differences in the number of NHX genes in plants. A total of 9 NHX genes have been identified in *C. sinensis* based on the Na^+^/H^+^ exchanger domain (Table 1).

*In-silico* studies based on subcellular localizations showed that NHXs are grouped into three classes (Vac-, Endo-, and PM-class). In *A. thaliana*, both NHX7 and NHX8 are localized in the plasma membrane (Shi et al., 2002), whereas in tea, *CsNHX9* (TEA006997.1) localized in the plasma membrane, *CsNHX8* (TEA011468.1) is localized in endosome, and the others in the vacuole (Table 1). Members in each of the classes from algae to higher plants showed that the NHX families were fairly similar, indicating that NHXs had conserved functions throughout the evolutionary process (Chanroj et al., 2012). The function of NHX transporters may be influenced by their subcellular localization. Members of the NHX family, which are found on both the plasma membrane and tonoplast, help to maintain ionic homeostasis by excluding and compartmentalizing excess Na^+^. Furthermore, endomembrane-bound NHX members have been discovered to be important for cellular cargo trafficking, growth development, and protein processing regulation (Bassil et al., 2011a,b). The exon/intron structural diversity, which plays an important role in the evolution of gene families, brings to the evidence for phylogenetic groupings. In *C. sinensis*, *CsNHX3* (TEA021179.1) possesses a greater number of introns (18) and exons (19) while *CsNHX1* (TEA012938.1) has lesser number of introns (12) and exons (18) than the rest of the 5 genes present in Vac-class. However, in *P. trichocarpa*, Vac-class NHXs (PtNHX1-5) contain 14 exons and the Endo-class NHX (PtNHX6) has 22 exons, while the PM-class NHXs (PtNHX7 and PtNHX8) displays 23 exons (Tian et al., 2017). Similarly, for NHX genes in *G. max* (Gm), seven members of GmNHX contain 14–15 exons, whereas the rest three members have 20 exons (Chen et al., 2015). These findings suggested that NHX gene families in plants have a fair share of structural diversity.

The putative amiloride-binding site and membrane-spanning pore in the NHX gene families, which contain the amino acid sequence “FFIYLLPPI” (Bassil et al., 2012), have been found to be highly conserved (Brett et al., 2005; Rodriguez Rosales et al., 2009; Bassil et al., 2012). In the presence of the drug amiloride and/or its derivatives, this domain inhibits the cation/H^+^ exchange (Wu et al., 2011). In the motif study, the amiloride-binding site was found to be located in the N-terminus of motif 3 and it is found in 6 NHX genes of *C. sinensis* (Figure 2). The C-terminus of NHX proteins was diverse in contrast to the conserved N-terminus. Studies have shown that the deletion of the C-terminal hydrophilic region results in increased Na^+^/H^+^ transport activity, implying that the C-terminus is important not only for subcellular localization but also for transport activity regulation (Yamaguchi et al., 2003; Orlowski and Grinstein, 2007). The phylogenetic analysis indicated that the NHXs in *P. trichocarpa* (Tian et al., 2017), *S. bicolor* (Kumari et al., 2018), and *Beta vulgaris* (Wu et al., 2019b) showed three phylogenetic clusters based on their location in the cell; we found the same results for tea NHX transporters. So according to these findings, the NHX family genes have remained relatively conserved throughout evolution.

Cis-acting regulatory elements function as key molecular switches in transcriptional regulation of gene activities that control a variety of biological processes, such as hormonal response, abiotic stress response, and development (Ding et al., 2018; Verma et al., 2019). Hormones including ABA, ethylene, SA, and IAA play significant roles in plants development and stress response (Mishra et al., 2014; Li et al., 2019; Wang and Huang, 2019; Zhang and Li, 2019). In this study, cis-acting regulatory elements related to transcription factors were identified to be randomly distributed across the promotor region of the 9 tea NHXs (Supplementary Table S2). One ABA-responsive element (ABRE) has been discovered in 6 NHXs (*CsNHX1*, *CsNHX2*, *CsNHX4*, *CsNHX5*, *CsNHX6*, and *CsNHX8*) of *C. sinensis* (Supplementary Table S2). Whereas in poplar, one or two ABREs were observed (Tian et al., 2017). This analysis showed that NHX genes may play a role in the ABA signaling pathway. Furthermore, ARE (anaerobic induction), DRE (drought-responsive cis-acting element), LTR (low-temperature responsive element), MBS (drought response), and STRE (stress response) were identified as stress responsive regulatory elements in tea. Similarly, in PtNHXs from poplar and SbNHXs from *S. bicolor* are also found to contain similar elements (Kumari et al., 2018). The results indicated that the identified regulatory elements in this study aid in understanding their roles in various abiotic and biotic stress-related pathways.

Further to understand the distribution pattern of the tea NHXs, the genomic distribution mapping was performed. Tandem duplication events were absent across the tea NHXs (Figure 5). The duplication of genes increases the functional divergence, which is an essential factor in adaptability under changing environmental conditions (Conant and Wolfe, 2008). The dN/dS ratio indicates different selection pressure on genes throughout the evolutionary changes. Wang et al. (2019) found that positive selection of a gene during evolution increases its potential and transcription levels under stress conditions in *Triticum aestivum* and TaBT1. Whereas in tea, the dN/dS ratios provided conclusive evidence that strong purifying selection pressure existed during evolution, allowing a variety of factors to regulate the genes (Supplementary Table S3).

In plants, sodium-proton antiporters facilitate both Na^+^/H^+^ and K^+^/H^+^ exchanges, contributing to stress tolerance as well as K^+^ nutrition (Venema et al., 2002; Apse et al., 2003; Leidi et al., 2010). NHXs have been reported to enhance salinity tolerance in different species, such as *A. thaliana* (Shi et al., 2000), *B. vulgaris* (Xia et al., 2002), *S. lycopersicum* (Zhang and Blumwald, 2001; Rodríguez-Rosales et al., 2008), *Hordeum vulgare* (Vasekina et al., 2005), *Z. mays* (Zörb et al., 2005), *T. aestivum* (Brini et al., 2005), *G. max* (Li et al., 2006), *O. sativa* (Fukuda et al., 2011; Zeng et al., 2018), and *S. bicolor* (Kumari et al., 2018). The expression data for various tissues and stress conditions showed that the tea NHXs may be involved in developmental processes and abiotic stress responses. Our study revealed that in *C. sinensis*, the NHX genes express differentially in 8 different tissues (Supplementary Table S5). The different expression patterns in various tissues (Figure 8) indicated that the NHX gene family provides opportunities to break the functional constraint from the original gene during the course of evolution.

Based on data from other species, functional annotation and interaction analysis of NHX proteins can help us predict their potential regulatory roles. The electrochemical gradient of protons across tonoplasts, generated by two vacuolar H^+^-pumps, H^+^-ATPase, and H^+^-PPase, has been shown to drive the Vac-class NHXs (Brett et al., 2005; Bao et al., 2009; Wu et al., 2011). In this analysis, all the tea genes considered for building the interaction network, belongs to the Vac-class. By increasing cation accumulation, co-expression of ZxNHX and ZxVP1 genes can improve salt tolerance in transgenic plant species, such as *Lotus corniculatus* (Bao et al., 2014), Alfalfa (Bao et al., 2015), and sugar beet (Wu et al., 2015). These finding suggested that when plants were exposed to salt stress, Vac-class NHXs might work together to transport Na^+^ across tonoplasts. Calcineurin B-like (CBL) is well known for its ability to interact and modulate CBL-interacting protein kinases (CIPK), which then mediate Ca^+^ signal transduction (Yu et al., 2014; Tian et al., 2017). During the salinity response, CBL regulates NHX7 (SOS1) and CIPK mediates the Ca^2+^ signaling pathway (Miranda et al., 2017). A salt stress elicited Ca^2+^ signal activates a protein kinase complex consisting of CBL4 (SOS3) and CIPK24 (SOS2), and the complex then phosphorylates and activates the SOS1 protein to extrude Na^+^ out of the cell in *A. thaliana* under salt stress (Quintero et al., 2011). In transgenic tobacco, overexpression of SOS1 gene increased salt tolerance by maintaining a higher K^+^/Na^+^ ratio (Yue et al., 2012). In the current study, CLBs are hypothesized to interact with TEA006066.1, *CsNHX1* (TEA012938.1), *CsNHX2* (TEA012286.1), *CsNHX3* (TEA021179.1), and *CsNHX4* (TEA012245.1) but not with CIPK (Figure 7). Similarly, NHX7 (SOS1) interactions with CBLs were predicted in poplar (Tian et al., 2017) and *S. bicolor* (Bassil et al., 2011a). However, in the future, yeast two hybrid research will need to confirm these proteins interactions.

Ion transporters are important in many biological processes, including ion uptake and sequestration, energy provision, and cell expansion (Bassil and Blumwald, 2014). Previous studies in plants found that Na^+^/H^+^ antiporters as important members in transporters mediate the coupled exchange of Na^+^ or K^+^ for H^+^ in all cellular compartments (Qiu, 2012; Bassil and Blumwald, 2014). The NHX genes primarily use two proton pumps, the H^+^-ATP enzyme and H^+^-PPase, to produce H^+^ electrochemical gradients that transport Na^+^ from the cytoplasm to vacuoles or outside the cell, thereby maintain Na^+^ ion stability and avoiding the toxic effect of Na^+^ accumulation in cells (Munns, 2002; Sze and Chanroj, 2018).

Stress response analysis showed that each tea NHX genes were responsive to abiotic stresses of drought, cold, and salt. Under PEG treatment, the expression of *CsNHX1* (TEA012938.1), *CsNHX4* (TEA012245.1), TEA00066.1, *CsNHX7* (TEA023041.1), *CsNHX8* (TEA011468.1) reached the highest level at 12 h (Figure 10), and *CsNHX1*, *CsNHX7* and *CsNHX8* also responded to salt stress in varying degree, demonstrating these genes may be associated with salt and drought stress. MeJA was found to be linked to salt tolerance in few studies (Zhang et al., 2019; Zhao et al., 2019) and the expression data suggested the close relation of the NHXs toward the regulation of MeJA. Further in the study, the expression levels of *CsNHX1*, *CsNHX7*, and *CsNHX8* were significantly upregulated by various concentrations of NaCl over a 48 h period and 72 h period (Figure 11), and their expression levels under high-salt stress were relatively higher than those under either mild or moderate-salt stress. In *Reaumuria trigyna*, the expression levels of RtNHX1 in leaves showed an increase and reached a high level at 3 h, and then reduced after 6 h when exposed to high-salt stress (200 mm NaCl; Li et al., 2017). A similar expression pattern was found in sweet potato, where IbNHX2 was significantly upregulated at 4 h after treatment of 200 mm NaCl (Wang et al., 2016). Another study (Lu et al., 2014) found that the transcription level of TaNHX3 in both leaves and roots sharply increased at 24 h and then gradually decreased after 48 h over a 96 h period in different wheat cultivars subjected to salt stress. Moreover, *CsNHX1* belonging to the Vac-class NHX showed the highest level of expression for all the salt stress condition. The study showed that the expression levels of Vac-class NHXs are significantly higher than other class genes thereby confirming that Vac-class NHXs might play critical roles in salt tolerance. The study also notices that *CsNHX1* and *CsNHX7* showed significant expression levels under all abiotic stress conditions thereby providing a comprehensive understanding of the functions of NHXs in *C. sinensis*.

## Conclusion

Among the numerous transporters in monovalent cation/proton antiporter (CAP1) family, the Na^+^/H^+^ antiporters (NHXs) are secondary ion transporters to exchange H^+^ and transfer the Na^+^ or K^+^ across membrane. The objective of this study was to identify, characterize, and determine the role of NHX genes in tea at the genomic stage. Using phylogenetic relationship, the 9 tea NHXs were grouped into three major classes (Vac-, Endo-, and PM-classes). The amiloride-binding site (FFIYLLPPI) is a characteristic feature of NHX proteins and it was found in the N-terminus of motif 3. The lack of tandem duplication events was a result of the close distribution pattern of the NHX genes in tea. ABA-responsive element (ABRE) was found in 6 genes, implying NHX gene might be involved in ABA signaling pathway as well. Furthermore, responses of tea NHX to drought, cold, and salinity indicated that the genes were involved in single or multiple stress responses. The study also confirms the active role of Vac-class NHXs in response to salt stress over the other two classes. *CsNHX1* (TEA012938.1) and *CsNHX7* (TEA023041.1) maintained high expression levels for all the abiotic stress conditions thereby giving us a comprehensive understanding of the role of NHX genes. Adding to the tally, the responses to MeJA treatment also suggested the involvement of the tea NHXs in MeJA regulation. Tea is a commercial crop, grown all over the world and abiotic stresses are one of the major factors that limit the crop productivity worldwide. This work will therefore serve as a basis to provide valuable information for future studies and exploration of the role of NHX genes in various developmental process, as well as the elucidation of other potential functions in *C. sinensis*.

## Data Availability Statement

The datasets presented in this study can be found in online repositories. The names of the repository/repositories and accession number(s) can be found in the article/Supplementary Material.

## Author Contributions

AP and AC designed and performed the experiments. SS and NM devised the experiments. GS helped in data analysis and writing the manuscript. All authors contributed to the article and approved the submitted version.

## Funding

This project was supported by the Key Technologies R&D Program for Crop Breeding of Zhejiang Province (2016C02054-19 and 2017C02010), the Natural Science Foundation of China (31670303), and the Joint Laboratory of Olive Oil Quality and Nutrition among China, Australia, and Spain.

## Conflict of Interest

The authors declare that the research was conducted in the absence of any commercial or financial relationships that could be construed as a potential conflict of interest.

## Publisher’s Note

All claims expressed in this article are solely those of the authors and do not necessarily represent those of their affiliated organizations, or those of the publisher, the editors and the reviewers. Any product that may be evaluated in this article, or claim that may be made by its manufacturer, is not guaranteed or endorsed by the publisher.

## Footnotes

^1^ http://tpia.teaplant.org/

^2^ https://www.arabidopsis.org/

^3^ http://rice.plantbiology.msu.edu/

^4^ http://smart.embl-heidelberg.de/

^5^ https://expasy.org/

^6^ http://gpcr.biocomp.unibo.it/bacello/index.htm

^7^ http://www.cbs.dtu.dk/services/TMHMM/

^8^ http://meme-suite.org/

^9^ http://gsds.cbi.pku.edu.cn/

^10^ http://bioinformatics.psb.ugent.be/webtools/plantcare/html/

^11^ http://mg2c.iask.in/mg2c_v2.0/

^12^ https://www.hiv.lanl.gov/content/sequence/SNAP/SNAP.html

^13^ https://www.ebi.ac.uk/

^14^ https://string-db.org/

## Abbreviations

ABA: Abscisic acid
ABRE: ABA-responsive element
ARE: Anaerobic-responsive element
CBL: Calcineurin B-like proteins
CDC2: Cell division cycle protein 2 CDS Coding sequences
CIPK: CBL-interacting protein kinases
CMP: Calcium-binding protein
CYB5R1: NADH-cytochrome b5 reductase 1
DER: Drought-responsive element
ERE: Ethylene-responsive element
GSDS: Gene structure display serve
GSK3: Glycogen synthase kinase 3
HKT: High-affinity K^+^ transporter
IAA: Indole Acetic Acid
LTR: Low-temperature responsiveness
ORF: Open reading frame
pI: Isoelectric point
PM: Plasma membrane
PPI: Protein–protein interaction
SA: Salicylic acid
SOS1: Salt overly sensitive 1
3-D: Three-dimension
TM: Transmembrane helical domain
VP: Vacuolar H^+^-PPase
MEME: Multiple expectation maximization for motif elicitation
MW: Molecular weight
NHX: Na^+^/H^+^ antiporter
ROS: Reactive-oxygen species
TPIA: Tea Plant Information Archive
CS/Cs: *Camellia sinensis*
TAIR: The Arabidopsis Information Resource
HMM: Hidden Markov Model
dN: non-synonymous substitution
dS: Synonymous substitution
NJ: Neighbor Joining
AT/At: *Arabidopsis thaliana*
CK: Non acclimated
CA1: Fully acclimated
CA3: De-acclimated
TPM: Transcripts per million
UTR: Untranslated Region
