# Supplementary material for: NHX Gene Family in Camellia sinensis: In-silico Genome-Wide Identification, Expression Profiles, and Regulatory Network Analysis
Source: Front Plant Sci. 2021 Dec 20;12:777884. doi: 10.3389/fpls.2021.777884 (PMC8720784; doi:10.3389/fpls.2021.777884)
Supplement: Supplementary file 1 [file Data_Sheet_1.pdf]

Additional File 1

NHX gene family in *Camelia sinensis*: *in-silico* genome wide identification, expression profiles and regulatory network analysis

Abhirup Paul[1*], Archita Chatterjee[1*], Shreya Subrahmanya[2], Guoxin Shen[3†], Neelam Mishra[2†]

[1]Independent researcher
Bangalore, Karnataka,
India

[2]Department of Botany
St. Joseph's College autonomous
Bangalore, Karnataka,
India

[3]Sericultural Research Institute,
Zhejiang Academy of Agricultural Sciences
Hangzhou 310021, China

*These authors contributed equally to this work.

†Corresponding authors:
Guoxin Shen, Ph.D., Professor, Tel: +86-571-86404298; Fax: +86-571-86404298
Email address: guoxin.shen@ttu.edu
Neelam Mishra, Ph.D., Assistant professor
Email address: neelamiitkgp@gmail.com; neelammishra@sjc.ac.in

Table S1. BLAST positives table for NHX genes in *C. sinensis*. Protein sequences from Arabidopsis genome database (TAIR) were taken and subjected to BLASTp in the Tea genome database (TPIA).

Arabidopsis NHX gene IDs	Tea NHX gene IDs	Identities	Positives	Gaps
**AT5G27150.1** (AtNHX1)	TEA012938.1	74%	85%	0%
	TEA012286.1	73%	84%	0%
	TEA021179.1	75%	87%	2%
	TEA012245.1	63%	76%	10%
	TEA000661.1	64%	76%	12%
	TEA025916.1	65%	79%	1%
**AT3G05030.1** (AtNHX2)	TEA012938.1	76%	86%	1%
	TEA012286.1	75%	85%	1%
	TEA021179.1	76%	87%	3%
	TEA012245.1	66%	75%	10%
	TEA000661.1	65%	76%	12%
	TEA025916.1	64%	79%	1%
**AT5G55470.1** (AtNHX3)	TEA012938.1	60%	75%	2%
	TEA012286.1	58%	73%	2%
	TEA023041.1	58%	69%	17%
	TEA025916.1	58%	74%	3%
	TEA021179.1	59%	75%	3%
	TEA012245.1	53%	65%	12%
**AT3G06370.1** (AtNHX4)	TEA012938.1	75%	83%	3%
	TEA012286.1	74%	83%	0%
	TEA021179.1	73%	83%	3%
	TEA012245.1	61%	72%	12%
	TEA000661.1	58%	71%	12%
	TEA025916.1	66%	81%	1%
**AT1G54370.1** (AtNHX5)	TEA012938.1	31%	50%	8%
**AT1G79610.1** (AtNHX6)	TEA011468.1	67%	76%	16%
**AT2G01980.1** (AtNHX7)	TEA006997.1	62%	75%	4%
**AT1G14660.1** (AtNHX8)	TEA006997.1	68%	81%	1%

Table S2. Function specific list of cis-acting elements. These elements have been identified from 2 kbp upstream region of all the identified NHX genes of *C. sinensis*.

Sl. no.	Cis-acting element identified	Sequence	Tea genes	Specific function of the cis element
1.	A-box	CCGTCC	TEA012938.1/ TEA006997.1	cis-acting regulatory element
2.	ABRE	CGTACGTGCA/ACGTG/CACGTG	TEA012938.1/ TEA012286.1/ TEA012245.1/ TEA000661.1/ TEA025916.1/ TEA011468.1	abscisic acid responsiveness
3.	ARE	AAACCA	TEA012938.1/ TEA012286.1/ TEA021179.1/ TEA012245.1/ TEA000661.1/ TEA025916.1/ TEA011468.1	anaerobic induction
4.	Box-4	ATTAAT	TEA012938.1/ TEA012286.1/ TEA021179.1/ TEA012245.1/ TEA000661.1/ TEA025619.1/ TEA011468.1/ TEA006997.1	light responsiveness
5.	CCAAT-box	CAACGG	TEA012938.1/ TEA021179.1/ TEA000661.1/ TEA011468.1/ TEA006997.1	MYBHv1 binding site
6.	G-box	TACGTG/tgACACGTGGCA/ CACGTT/ TCCACATGGCA/ CACGAC/ GCCACGTGGA / CTTCCACGTGGCA	TEA012938.1/ TEA012286.1/ TEA012245.1/ TEA000661.1/ TEA025619.1/ TEA011468.1	light responsiveness
7.	GARE-motif	TCTGTTG	TEA012938.1/ TEA000661.1	gibberellin-responsive element
8.	TCT-motif	TCTTAC	TEA012938.1	light responsive element
9.	chs-CMA2a	TCACTTGA	TEA012938.1/ TEA012286.1/ TEA011468.1	light responsive element
10.	AE-box	AGAAACAA	TEA012286.1/ TEA023041.1	light response
11.	CGTCA-motif	CGTCA	TEA012286.1/ TEA012245.1/ TEA025619.1/ TEA011468.1	MeJA-responsiveness
12.	LTR	CCGAAA	TEA012286.1/ TEA012245.1	low-temperature responsiveness
13.	MRE	AACCTAA	TEA012286.1/ TEA000661.1/ TEA011468.1	light responsiveness
14.	O2-site	GATGA(C/T)(A/G)TG(A/G)	TEA012286.1/ TEA012245.1/ TEA011468.1/ TEA006997.1	zein metabolism regulation
15.	TCCC-motif	TCTCCCT	TEA012286.1/ TEA021179.1/ TEA012245.1/ TEA000661.1	light responsive element
16.	TGACG-motif	TGACG	TEA012286.1/ TEA012245.1/ TEA025619.1/ TEA011468.1	MeJA-responsiveness
17.	GATA-motif	GATAGGG/ AAGATAAGATT	TEA021179.1/ TEA012245.1/ TEA025619.1/ TEA023041.1/ TEA006997.1	light responsive element
18.	GCN4_motif	TGAGTCA	TEA021179.1/ TEA000661.1/ TEA023041.1	endosperm expression
19.	Gap-box	CAAATGAA(A/G)A	TEA021179.1	light responsive element
20.	HD-Zip 1	CAAT(A/T)ATTG	TEA021179.1/ TEA023041.1/ TEA006997.1	differentiation of the palisade mesophyll cells
21.	I-box	gGATAAGGTG	TEA021179.1	light responsive element
22.	TATC-box	TATCCCA	TEA021179.1/ TEA000661.1/ TEA006997.1	gibberellin-responsiveness
23.	AAAC-motif	CAATCAAAACCT	TEA012245.1	light responsive element
24.	TC-rich repeats	GTTTTCTTAC	TEA012245.1/ TEA025619.1/ TEA011468.1	defense and stress responsiveness
25.	TCA-element	CCATCTTTTT	TEA012245.1/ TEA000661.1/ TEA025619.1/ TEA023041.1/ TEA011468.1	salicylic acid responsiveness
26.	TCT-motif	TCTTAC	TEA012245.1/ TEA025619.1/ TEA023041.1/ TEA011468.1	light responsive element
27.	chs-CMA1a	TTACTTAA	TEA012245.1/ TEA025619.1/ TEA006997.1	light responsive element
28.	CAT-box	GCCACT	TEA000661.1	meristem expression
29.	MBS	CAACTG	TEA000661.1	drought-inducibility
30.	TGA-element	AACGAC	TEA000661.1	auxin-responsive element

31.	Circadian	CAAAGATATC	TEA000661.1	circadian control
32.	AT1-motif	AATTATTTTTTATT	TEA025619.1/ TEA006997.1	light responsive element
33.	Box II	CCACGTGGC	TEA025619.1	light responsive element
34.	GT1-motif	GGTTAA	TEA025619.1/ TEA023041.1/ TEA011468.1	light responsive element
35.	AT-rich sequence	TAAAATACT	TEA023041.1	maximal elicitor-mediated activation
36.	AuxRR-core	GGTCCAT	TEA023041.1	auxin responsiveness
37.	GA-motif	ATAGATAA	TEA023041.1	light responsive element
38.	P-box	CCTTTTG	TEA023041.1/ TEA006997.1	gibberellin-responsive element
39.	AT-rich element	ATAGAAATCAA	TEA011468.1/ TEA006997.1	binding site of AT-rich DNA binding protein
40.	ATCT-motif	AATCTAATCC	TEA011468.1	Light responsiveness
41.	MSA-like	(T/C)C(T/C)AACGG(T/C)(T/C)A	TEA011468.1	ell cycle regulation

Table S3. dN/dS ratios of 9 NHX genes in *C. sinensis*. SNAP v2.1.1 Synonymous Non-synonymous Analysis Program was used to analyse the dN/dS ratios. This ratio is useful to understand the positive or negative selection of the genes.

Compare	Sequence names	Sd	Sn	S	N	ps	pn	ds	dn	dn/ds	ds/dn	ps/pn
0 1	TEA012938.1 TEA012286.1	862.8333	3382.167	1220.833	4692.167	0.7068	0.7208	2.1399	2.4347	1.1378	0.8789	0.9805
0 2	TEA012938.1 TEA021179.1	1462.167	5786.833	2061.167	7991.833	0.7094	0.7241	2.187	2.5242	1.1542	0.8664	0.9797
0 3	TEA012938.1 TEA012245.1	1266.5	4946.5	1758.333	6767.667	0.7203	0.7309	2.4213	2.7529	1.137	0.8796	0.9855
0 4	TEA012938.1 TEA000661.1	1384.333	5460.667	1960.333	7522.667	0.7062	0.7259	2.1299	2.5782	1.2105	0.8261	0.9728
0 5	TEA012938.1 TEA025916.1	1086.333	4473.667	1563	6216	0.695	0.7197	1.96	2.4067	1.228	0.8144	0.9657
0 6	TEA012938.1 TEA023041.1	1531.333	5795.667	2130.667	7922.333	0.7187	0.7316	2.3826	2.7792	1.1664	0.8573	0.9824
0 7	TEA012938.1 TEA011468.1	488.6667	1809.333	674.6667	2520.333	0.7243	0.7179	2.5304	2.3633	0.9334	1.0707	1.0089
0 8	TEA012938.1 TEA006997.1	1480	5750	2068.333	7984.667	0.7156	0.7201	2.3105	2.4174	1.0463	0.9558	0.9936
1 2	TEA012286.1 TEA021179.1	870	3498	1234.833	4678.167	0.7045	0.7477	2.1026	4.3498	2.0688	0.4834	0.9423
1 3	TEA012286.1 TEA012245.1	914	3416	1247.167	4665.833	0.7329	0.7321	2.834	2.8027	0.989	1.0112	1.001
1 4	TEA012286.1 TEA000661.1	919.5	3375.5	1238.667	4674.333	0.7423	0.7221	3.4371	2.4695	0.7184	1.3918	1.028
1 5	TEA012286.1 TEA025916.1	885.1667	3391.833	1210.5	4702.5	0.7312	0.7213	2.7663	2.4469	0.8845	1.1305	1.0138
1 6	TEA012286.1 TEA023041.1	907.6667	3372.333	1244.833	4668.167	0.7291	0.7224	2.6869	2.477	0.9107	1.0848	1.0093
1 7	TEA012286.1 TEA011468.1	495.3333	1821.667	685.5	2509.5	0.7226	0.7259	2.4818	2.5787	1.039	0.9624	0.9954
1 8	TEA012286.1 TEA006997.1	900.5	3348.5	1231.167	4681.833	0.7314	0.7152	2.7735	2.3031	0.8304	1.2043	1.0227
2 3	TEA021179.1 TEA012245.1	1227.167	4960.833	1751.333	6774.667	0.7007	0.7323	2.0417	2.8083	1.3755	0.727	0.9569
2 4	TEA021179.1 TEA000661.1	1366	5457	1943.333	7539.667	0.7029	0.7238	2.0761	2.5149	1.2113	0.8255	0.9712
2 5	TEA021179.1 TEA025916.1	1135.833	4443.167	1571.167	6207.833	0.7229	0.7157	2.4911	2.3145	0.9291	1.0763	1.01
2 6	TEA021179.1 TEA023041.1	2594.667	9668.333	3561.833	13394.17	0.7285	0.7218	2.6628	2.4614	0.9244	1.0818	1.0092
2 7	TEA021179.1 TEA011468.1	505.3333	1832.667	691.1667	2503.833	0.7311	0.7319	2.7619	2.795	1.0119	0.9882	0.9989
2 8	TEA021179.1 TEA006997.1	3142.833	12411.17	4396.667	17074.33	0.7148	0.7269	2.2947	2.6099	1.1374	0.8793	0.9834
3 4	TEA012245.1 TEA000661.1	1215.667	4763.333	1763.333	6762.667	0.6894	0.7044	1.887	2.0994	1.1125	0.8988	0.9788
3 5	TEA012245.1 TEA025916.1	1168.333	4496.667	1583	6196	0.7381	0.7257	3.1045	2.5733	0.8289	1.2064	1.017

3 6	TEA012245.1 TEA023041.1	1302	4904	1789.167	6736.833	0.7277	0.7279	2.6371	2.6447	1.0029	0.9971	0.9997
3 7	TEA012245.1 TEA011468.1	493.3333	1831.667	693.5	2501.5	0.7114	0.7322	2.2245	2.8068	1.2618	0.7925	0.9715
3 8	TEA012245.1 TEA006997.1	1292.333	4860.667	1768.5	6757.5	0.7308	0.7193	2.747	2.3968	0.8725	1.1461	1.0159
4 5	TEA000661.1 TEA025916.1	1115	4483	1577	6202	0.707	0.7228	2.1448	2.4885	1.1602	0.8619	0.9782
4 6	TEA000661.1 TEA023041.1	1421.5	5444.5	2002.167	7480.833	0.71	0.7278	2.198	2.6398	1.201	0.8327	0.9755
4 7	TEA000661.1 TEA011468.1	485	1828	678.5	2516.5	0.7148	0.7264	2.2945	2.5943	1.1307	0.8844	0.984
4 8	TEA000661.1 TEA006997.1	1383.667	5404.333	1958.667	7524.333	0.7064	0.7182	2.1343	2.3716	1.1112	0.9	0.9836
5 6	TEA025916.1 TEA023041.1	1162	4411	1602	6177	0.7253	0.7141	2.5613	2.2795	0.89	1.1236	1.0157
5 7	TEA025916.1 TEA011468.1	478.6667	1808.333	668.6667	2526.333	0.7159	0.7158	2.317	2.3157	0.9994	1.0006	1.0001
5 8	TEA025916.1 TEA006997.1	1109.833	4434.167	1583	6196	0.7011	0.7156	2.0476	2.3126	1.1294	0.8854	0.9797
6 7	TEA023041.1 TEA011468.1	504.1667	1813.833	689.8333	2505.167	0.7309	0.724	2.7509	2.5226	0.917	1.0905	1.0094
6 8	TEA023041.1 TEA006997.1	2623	9721	3612	13344	0.7262	0.7285	2.5875	2.6637	1.0294	0.9714	0.9968
7 8	TEA011468.1 TEA006997.1	455.8333	1843.167	670.5	2524.5	0.6798	0.7301	1.777	2.7225	1.5321	0.6527	0.9311

Table S4. The GO enrichment analysis of all the 9 NHX genes of *C. sinensis*. The potential functions of the genes are divided in 3 major categories based on their biological function, cellular component and molecular function.

**BIOLOGICAL PROCESS**			
**GO Ids**	**Description**	**Frequency of genes**	**Genes**
GO:0055067	Monovalent inorganic cation homeostasis	3	TEA012938.1, TEA012286.1, TEA025916.1
GO:1902600	Proton transmembrane transport	6	TEA012938.1, TEA012286.1, TEA021179.1, TEA012245.1, TEA000661.1, TEA006997.1
GO:0035725	Sodium ion transmembrane transport	5	TEA012938.1, TEA012286.1, TEA021179.1, TEA012245.1, TEA000661.1
GO:0055075	Potassium ion homeostasis	5	TEA012938.1, TEA012286.1, TEA021179.1, TEA012245.1, TEA000661.1
GO:0006885	Regulation of pH	5	TEA012938.1, TEA012286.1, TEA021179.1, TEA012245.1, TEA000661.1
GO:0009651	Response to salt stress	5	TEA012938.1, TEA012286.1, TEA021179.1, TEA012245.1, TEA000661.1
GO:0030001	Metal ion transport	3	TEA012938.1, TEA012286.1, TEA025916.1
GO:0042794	rRNA transcription from plastid promoter	1	TEA021179.1
GO:0006355	Regulation of transcription, DNA-templated	1	TEA021179.1
GO:0008380	RNA splicing	1	TEA021179.1
GO:0009793	Embryo development ending in seed dormancy	1	TEA021179.1
GO:0006812	Cation transport	2	TEA012245.1, TEA006997.1
GO:0006811	Ion-transport	1	TEA000661.1
**CELLULAR COMPONENT**			
**GO Ids**	**Description**	**Number of genes**	**Genes**
GO:0031224	Intrinsic component of membrane	3	TEA012938.1, TEA012286.1, TEA025916.1

GO:0005774	Vacuolar membrane	5	TEA012938.1, TEA012286.1, TEA021179.1, TEA012245.1, TEA000661.1
GO:0016021	Integral component of membrane	6	TEA012938.1, TEA012286.1, TEA021179.1, TEA012245.1, TEA000661.1, TEA006997.1
GO:0005886	Plasma membrane	5	TEA012938.1, TEA012286.1, TEA021179.1, TEA012245.1, TEA000661.1
GO:0009536	Plastid	1	TEA021179.1
GO:0005739	Mitochondrion	1	TEA021179.1
**MOLECULAR FUNCTION**			
**GO Ids**	**Description**	**Number of genes**	**Genes**
GO:0005451	Monovalent cation:proton antiporter activity	3	TEA012938.1, TEA012286.1, TEA025916.1
GO:0015385	Sodium:proton antiporter activity	5	TEA012938.1, TEA012286.1, TEA021179.1, TEA012245.1, TEA000661.1
GO:0003690	Double-stranded DNA binding	1	TEA021179.1
GO:0015299	Solute:proton antiporter activity	2	TEA012245.1, TEA006997.1
GO:0015297	Antiporter activity	1	TEA000661.1

Table S5. Tissue specific expression data of tea NHXs

Gene	Apical bud	Flower	Fruit	Young leaf	Mature leaf	Old leaf	Root	Stem
**TEA012938.1**	43.76413	121.8748	139.7705	65.48526	62.31895	89.1654	123.3367	89.58724
**TEA012286.1**	13.32334	19.94589	81.18094	19.25734	195.6898	154.8273	20.42718	38.48513
**TEA021179.1**	6.507971	5.212446	6.059097	6.496247	18.67248	11.14241	3.025886	6.226808
**TEA012245.1**	1.826799	3.354445	3.280869	2.031149	5.085746	1.680067	5.336918	6.342119
**TEA000661.1**	39.60114	52.39562	30.45085	29.85017	45.4933	79.72917	108.1191	44.66389
**TEA025916.1**	11.08375	9.490869	8.965271	6.847627	18.37551	12.92694	0.793193	11.76175
**TEA023041.1**	0.202002	0.281211	0.027417	0.239967	0.296978	0.374315	0.19585	0
**TEA011468.1**	3.117854	10.61571	6.342403	3.530942	6.570635	6.537461	4.768953	6.447821
**TEA006997.1**	3.776556	4.35877	7.137489	4.473669	6.041643	1.706182	8.539069	10.8873

Table S6. Expression data of tea NHXs under cold stress

Gene	CK	CA1-6h	CA1-7d	CA2-7d	DA-7d
**TEA012938.1**	29.45019	72.85738	75.17316	96.87901	55.52682
**TEA012286.1**	126.4097	131.4313	180.7668	107.6854	73.9297
**TEA021179.1**	16.90728	19.83296	28.62055	22.15093	15.32244
**TEA012245.1**	3.33917	3.08202	1.960717	2.282841	1.012626
**TEA000661.1**	50.84357	81.24412	74.82189	118.5099	39.75186
**TEA025916.1**	18.55783	12.11618	18.69256	15.13589	12.69169
**TEA023041.1**	0.037597	0.119941	0.163987	0.041164	0
**TEA011468.1**	7.288367	8.120182	14.04962	12.14041	12.64389
**TEA006997.1**	8.882562	11.54806	6.637157	3.834754	15.04385

Table S7: Expression data of the tea NHXs under drought stress

Gene	N-0h	PEG-N-24h	PEG-N-48h	PEG-N-72h
**TEA012938.1**	38.15789	106.9188	97.85543	165.0236
**TEA012286.1**	75.64702	24.13241	61.51073	28.75782
**TEA021179.1**	10.77421	3.648387	6.541141	3.283459
**TEA012245.1**	1.336108	2.915556	2.109869	1.71389
**TEA000661.1**	40.86235	69.21883	59.24442	81.5388
**TEA025916.1**	8.871773	3.104651	4.656081	2.715445
**TEA023041.1**	0	0.558879	0.357562	0.458908
**TEA011468.1**	6.333739	15.93189	16.78334	17.68513
**TEA006997.1**	2.21532	1.140223	1.441681	1.182608

Table S8: Expression data of the tea NHXs under salt stress

Gene	N-0h	NaCl-N-24h	NaCl-N-48h	NaCl-N-72h
**TEA012938.1**	38.1579	72.25577	99.93413	192.6962
**TEA012286.1**	75.64704	23.65101	35.82729	7.036825
**TEA021179.1**	10.77421	5.110744	5.70018	3.065808
**TEA012245.1**	1.336108	0.951984	1.237334	0.879937
**TEA000661.1**	40.86238	22.79735	32.173	22.55661
**TEA025916.1**	8.871778	1.774618	3.725586	2.337885
**TEA023041.1**	0	0.066448	0.458791	0.211543
**TEA011468.1**	6.333737	15.48246	15.18481	9.151531
**TEA006997.1**	2.21532	1.802493	1.944796	0.327543

Table S9. Expression data of the tea NHXs under MeJA treatment

Gene	CK	12h_MeJA	24h_MeJA	48h_MeJA
**TEA012938.1**	34.19849	41.5685	59.2445	51.77012
**TEA012286.1**	42.00289	59.01399	41.90896	45.30166
**TEA021179.1**	14.52917	13.38986	13.45105	12.41783
**TEA012245.1**	1.586705	2.463537	2.125546	1.986136
**TEA000661.1**	42.73238	48.82838	44.49009	75.10711
**TEA025916.1**	15.31124	12.53217	15.59798	17.83681
**TEA023041.1**	0.041174	0.095074	0.07173	0
**TEA011468.1**	15.59616	23.34664	22.99138	11.62203
**TEA006997.1**	11.24856	13.71702	13.93264	8.334638
