# Supplementary material for: NHX Gene Family in Camellia sinensis: In-silico Genome-Wide Identification, Expression Profiles, and Regulatory Network Analysis
Source: Front Plant Sci. 2021 Dec 20;12:777884. doi: 10.3389/fpls.2021.777884 (PMC8720784; doi:10.3389/fpls.2021.777884)
Supplement: Supplementary file 2 [file Data_Sheet_2.pdf]

Additional File 2

NHX gene family in *Camelia sinensis*: *in-silico* genome wide identification, expression profiles and regulatory network analysis

Abhirup Paul[1*], Archita Chatterjee[1*], Shreya Subrahmanya[2], Guoxin Shen[3†], Neelam Mishra[2†]

[1]Independent researcher
Bangalore, Karnataka,
India

[2]Department of Botany
St. Joseph's College autonomous
Bangalore, Karnataka,
India

[3]Sericultural Research Institute,
Zhejiang Academy of Agricultural Sciences
Hangzhou 310021, China

*These authors contributed equally to this work.

†Corresponding authors:
Guoxin Shen, Ph.D., Professor, Tel: +86-571-86404298; Fax: +86-571-86404298
Email address: guoxin.shen@ttu.edu
Neelam Mishra, Ph.D., Assistant professor
Email address: neelamiitkgp@gmail.com; neelammishra@sjc.ac.in

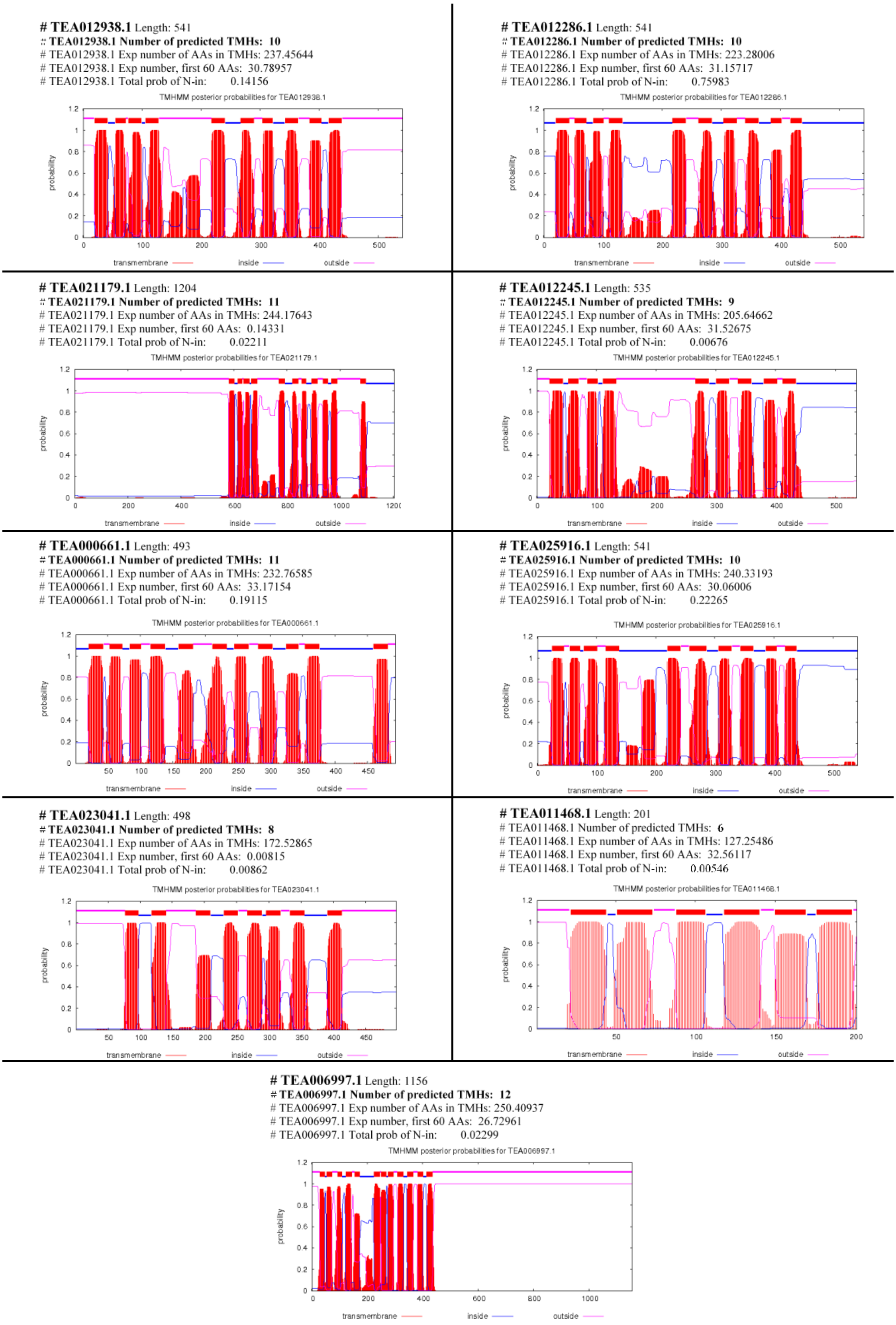

Figure S1. Transmembrane helices of NHX genes in *C. sinensis*. TMHMM Server, v.2.0 (http://www.cbs.dtu.dk/services/TMHMM/), was used to generate the transmembrane helices.

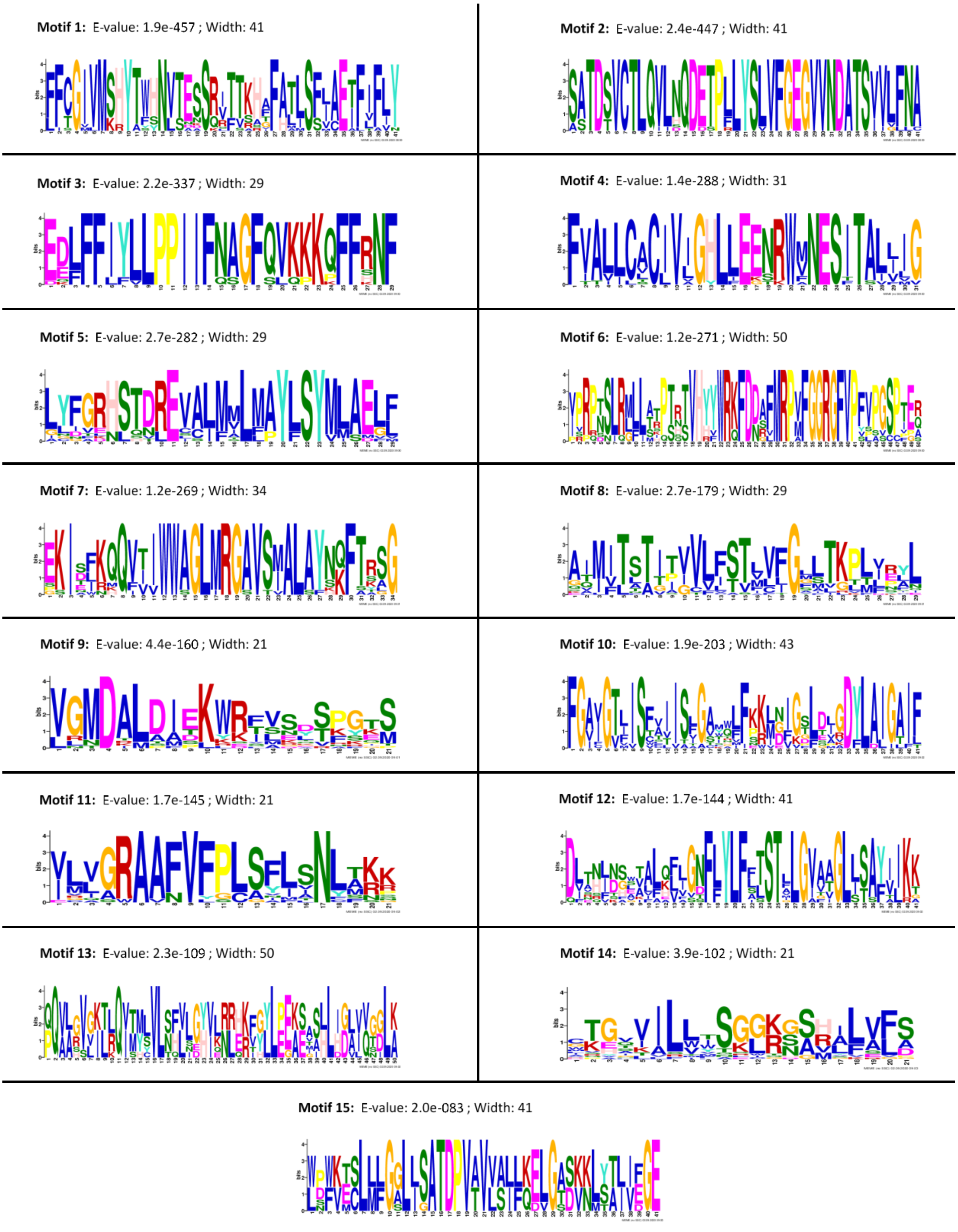

Figure S2. Motif logos of the 15 identified motifs in 24 NHX genes from *Arabidopsis thaliana*, *C. sinensis* and *Oryza sativa*. The motif logos were generated by MEME suite.

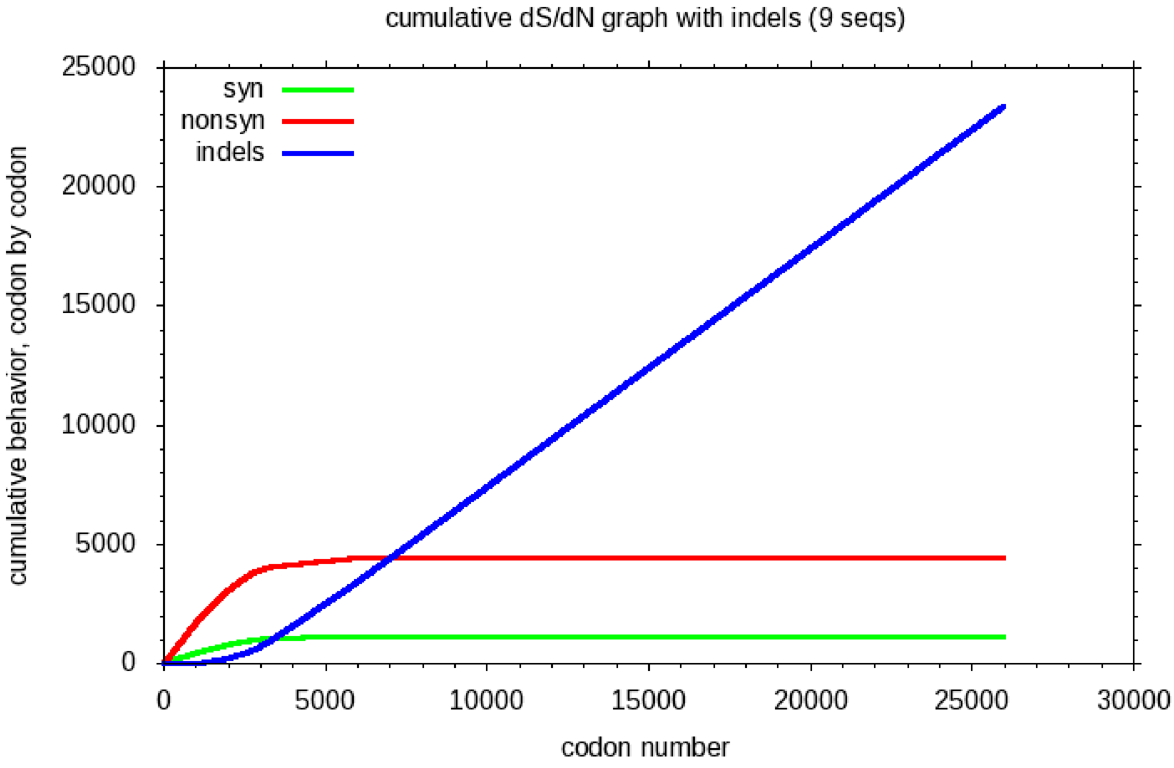

Figure S3. The ds/dn cumulative graph of NHX genes in *C. sinensis*. SNAP server (https://www.hiv.lanl.gov/content/sequence/SNAP/SNAP.html) has been used to generate the graph.
